# Insights into the Serum Metabolic Adaptations in Response to Inspiratory Muscle Training: A Metabolomic Approach Based on ^1^H NMR and UHPLC-HRMS/MS

**DOI:** 10.3390/ijms242316764

**Published:** 2023-11-25

**Authors:** Alex Castro, Aparecida M. Catai, Patrícia Rehder-Santos, Étore F. Signini, Raphael Martins de Abreu, Claudio Donisete Da Silva, Carla Cristina Dato, Regina V. Oliveira, Antônio G. Ferreira

**Affiliations:** 1 Department of Chemistry, Universidade Federal de São Carlos (UFSCar), São Carlos 13565-905, Brazil; oliveirarv@ufscar.br; 2 Biosciences National Laboratory (LNBio), Brazilian Center for Research in Energy and Materials (CNPEM), Campinas 13083-100, Brazil; 3 Department of Physiotherapy, Universidade Federal de São Carlos (UFSCar), Sao Carlos 13565-905, Brazil; mcatai@ufscar.br (A.M.C.); rehderpaty@hotmail.com (P.R.-S.); efsignini@estudante.ufscar.br (É.F.S.); raphaelmartins.abreu@yahoo.com.br (R.M.d.A.); claudio_acrobatica@hotmail.com (C.D.D.S.); 4 Nutrition Course, Central Paulista University Center, Sao Carlos 13563-470, Brazil; carladatonutricionista@gmail.com

**Keywords:** metabolome, metabolism, omic sciences, breathing exercises, ^1^H NMR, LC-HRMS

## Abstract

Inspiratory muscle training (IMT) is known to promote physiological benefits and improve physical performance in endurance sports activities. However, the metabolic adaptations promoted by different IMT prescribing strategies remain unclear. In this work, a longitudinal, randomized, double-blind, sham-controlled, parallel trial was performed to investigate the effects of 11 weeks (3 days·week^−1^) of IMT at different exercise intensities on the serum metabolomics profile and its main regulated metabolic pathways. Twenty-eight healthy male recreational cyclists (30.4 ± 6.5 years) were randomized into three groups: sham (6 cm·H_2_O of inspiratory pressure, *n* = 7), moderate-intensity (MI group, 60% maximal inspiratory pressure (MIP), *n* = 11) and high-intensity (HI group, 85–90% MIP, n = 10). Blood serum samples were collected before and after 11 weeks of IMT and analyzed by ^1^H NMR and UHPLC-HRMS/MS. Data were analyzed using linear mixed models and metabolite set enrichment analysis. The ^1^H NMR and UHPLC-HRMS/MS techniques resulted in 46 and 200 compounds, respectively. These results showed that ketone body metabolism, fatty acid biosynthesis, and aminoacyl-tRNA biosynthesis were upregulated after IMT, while alpha linolenic acid and linoleic acid metabolism as well as biosynthesis of unsaturated fatty acids were downregulated. The MI group presented higher MIP, Tryptophan, and Valine levels but decreased 2-Hydroxybutyrate levels when compared to the other two studied groups. These results suggest an increase in the oxidative metabolic processes after IMT at different intensities with additional evidence for the upregulation of essential amino acid metabolism in the MI group accompanied by greater improvement in respiratory muscle strength.

## 1. Introduction

Sports performance in endurance athletes can be limited by various factors, primarily due to the impairment of the functioning of the respiratory system. The primary role of the respiratory system involves regulating alveolar ventilation while maintaining the oxygen (O_2_) uptake and carbon dioxide (CO_2_) exhalation according to the metabolic demands of the body [1]. This process prevents the excessive increase in arterial blood carbon dioxide pressure (PaCO_2_) and the decrease in arterial blood oxygen pressure (PaO_2_) [2]. Despite this, during a vigorous and prolonged exercise routine, the respiratory muscles experience increased workload and dyspnea. This fact leads to a reduction in the overall respiratory function and endurance due to accumulated fatigue of respiratory muscles [3,4], limiting their ability to supply oxygen to working muscles [5,6].

In this sense, studies have explored the potential benefits of inspiratory muscle training (IMT) on physiological parameters and athletes’ endurance performance. IMT is a low-cost, easy-to-apply, safe, and nonpharmacological intervention that focuses on enhancing the strength or endurance of the muscles involved in breathing, such as the diaphragm and accessory muscles of inspiration [3]. IMT is frequently used as an important component in cardiorespiratory rehabilitation programs in individuals with respiratory muscle weakness [7,8,9], as well as an alternative to improve the sports’ endurance performance of amateur and elite athletes, by promoting improved cardiac vagal autonomic control in a resting condition, lower autonomic response during orthostatic challenge [10], and reduced fatigue and sensations of dyspnea in these individuals [10,11].

Previous studies suggest that mechanisms underlying fatigue of respiratory muscles affect exercise performance via a phenomenon known as respiratory metaboreflex [12]. The metaboreflex is characterized by the accumulation of lactate and other metabolites in the respiratory muscles during high-intensity exertion, which triggers a peripheral sympathetic response promoting vasoconstriction in the exercise limbs and early exercise termination [13,14]. IMT may attenuate the metaboreflex by increasing the strength and endurance of respiratory muscles [15] and reducing perceived breathlessness, lactate accumulation, sympathetic activation [13], and peripheral fatigue while improving exercise endurance performance [5,16,17]. Although precedent literature is devoted to understanding the physiological adaptation to IMT [10,14,18,19], details regarding metabolic adaptive responses remain little known [19,20].

Toward this goal, metabolomics has emerged as an analytical tool for the systematic study of metabolites (i.e., amino acids, organic acids, carbohydrates, nucleotides, and lipids) in a biological sample, typically utilizing either proton nuclear magnetic resonance spectroscopy (^1^H NMR) or ultra-high performance liquid chromatography coupled to high-resolution mass spectrometry (UHPLC-HRMS/MS), to follow the metabolic processes in response to exercise training and determinants of sports performance [21,22].

To date, only a few studies have investigated the induced metabolic adaptations instigated by IMT based on metabolomic approaches [19,20]; moreover, their results are controversial. In a previous study of our group, we showed that 11 weeks of IMT at different intensities based on maximal inspiratory pressure (MIP) (moderate-intensity group (60% MIP) and high-intensity group (85–90% MIP)) did not change the serum metabolic profile in 34 apparently healthy recreational male cyclists using ^1^H NMR-based metabolomics [20]. On the other hand, Craighead et al. verified increased levels of plasma ornithine, arginine, indole, and hexanoic acid after 6 weeks of IMT at 55–75% MIP in 36 apparently healthy subjects (midlife/older adults; 50–79 years) using UHPLC-HRMS/MS-based metabolomics [19]. The discrepancy between these studies can partially be explained by the different experimental designs but mainly due to limited coverage of the metabolome obtained when using a single analytical platform (e.g., ^1^H NMR or UHPLC-HRMS/MS) and sensitivity differences among the two techniques, as well as due to particularities of the sample preparation protocols used.

In this way, the training-induced metabolic adaptation in athletes is expected to be sport- or activity-specific. For instance, endurance training tends to increase the activity of oxidative enzymes and mitochondrial content in muscle [22,23]. In the case of IMT, it is likely that the systematic work of the respiratory muscles is reflected in typical chronic metabolic adaptations of endurance exercises as it improves lipid, carbohydrate, and amino acid metabolism [22], which can likely be assessed when using a comprehensive multiplatform metabolomic approach [21,22,24,25]. The investigation of the IMT-induced metabolic adaptations can contribute to the identification of underlying metabolism mechanisms that have not yet been discovered, providing crucial information to further IMT prescriptions for athletes with greater precision [20,22].

Therefore, the aim of this study was to investigate the effects of IMT, at different exercise intensities, on systemic metabolic adaptations, highlighting the main regulated metabolic pathways in apparently healthy recreational male cyclists, employing a comprehensive metabolomic approach based on ^1^H NMR and UHPLC-HRMS/MS.

## 2. Results

### 2.1. Participants

The sham, moderate-intensity (MI) and high-intensity (HI) groups were comparable for age, height, body mass, body mass index (BMI), body fat, inspiratory muscle strength (IMS), and aerobic power ($\dot{V}$O_2peak_) at the baseline (*p* < 0.05 for all; Table 1). On the other hand, a significant group–time interaction was observed for IMS (*p* = 0.015). The IMS significantly increased after the IMT for sham (+15.9%; Pre: 149.9 ± 14.1 cm·H_2_O vs. Post: 173.7 ± 19.2 cm·H_2_O; *p* = 0.005), MI (+31.1%; Pre: 159.4 ± 24.8 cm·H_2_O vs. Post: 209.0 ± 34.7 cm·H_2_O; *p* < 0.001), and HI (+37.1%; Pre: 146.8 ± 12.8 cm·H_2_O vs. Post: 201.2 ± 20.4 cm·H_2_O; *p* < 0.001) groups. Additionally, the MI group presented higher values, while the HI group showed a trend to elevated values when compared to the sham group post IMT (*p* = 0.010 and *p* = 0.059, respectively). No changes were observed between before and after the IMT program for body mass and body fat mass for all groups (*p* > 0.05 for all).

### 2.2. Metabolomics Data

**Figure 1:**
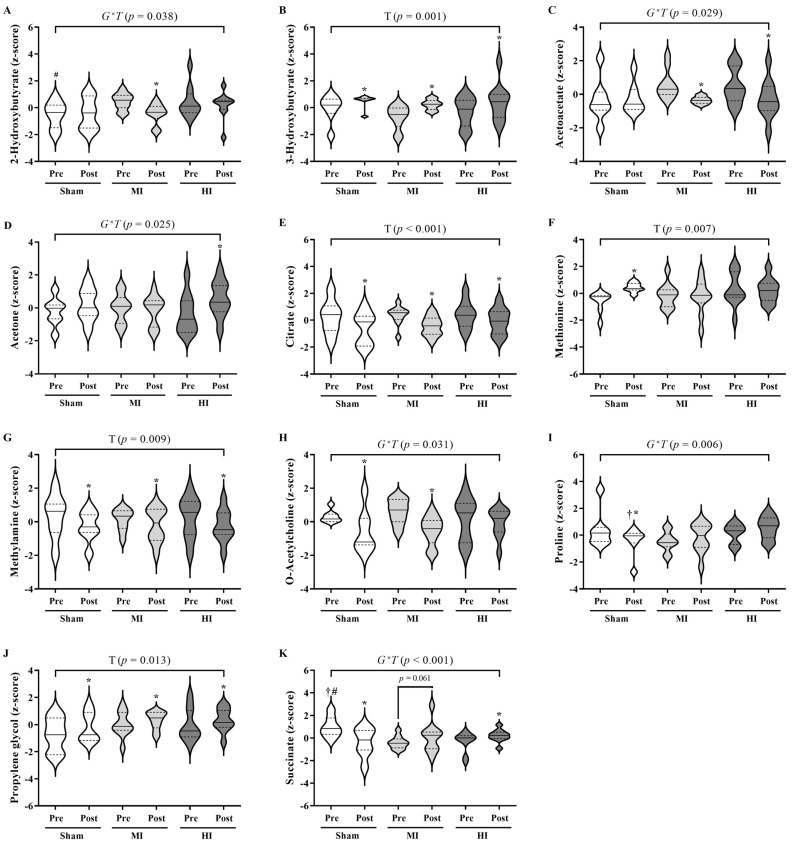
Violin plots for comparisons between and within groups of the annotated metabolites from ^1^H NMR-based metabolomics ((**A**): 2-Hydroxybutyrate, (**B**): 3-Hydroxybutyrate, (**C**): Acetoacetate, (**D**): Acetone, (**E**): Citrate, (**F**): Methionine, (**G**): Methylamine, (**H**): O-Acetylcholine, (**I**): Proline, (**J**): Propylene glycol, and (**K**): Succinate). Dashed lines represent the 1st and 3rd quartiles; T: Time main effect; G^×^T: group–time interaction; * difference from pre (*p* < 0.05); ^#^ difference from MI group (*p* < 0.05); ^†^ difference from HI group (*p* < 0.05); mixed linear model analyses were conducted using SPSS software version 25.0. The figure was generated using GraphPad Prism 9.

The ^1^H NMR and UHPLC-HRMS/MS techniques resulted in 46 and 200 serum putatively annotated compounds, respectively. The compounds that presented significant main effects or interactions in linear mixed models (*p* < 0.05 and a false discovery rate (FDR) of 0.2) were selected for further analysis (Table S1–S3) and are presented in detail in the Supplementary Materials (Figure 1 and Table S4).

For the ^1^H NMR-based metabolomics (Figure 1), all groups showed increased metabolic levels of 3-Hydroxybutyrate (*p* = 0.001) and Propylene glycol (*p* = 0.013), but decreased levels of Citrate (*p* < 0.001) and Methylamine (*p* = 0.009) post IMT. The sham group presented decreased levels of O-Acetylcholine (*p* < 0.004), Proline (*p* = 0.008), and Succinate (*p* < 0.001), but increased levels of Methionine (*p* = 0.001) post IMT. The MI group showed decreased levels of 2-Hydroxybutyrate (*p* = 0.001), Acetoacetate (*p* < 0.001) and O-Acetylcholine (*p* < 0.001), but a trend showing increasing levels of Succinate (*p* = 0.061) post IMT. The HI group showed decreased levels of Acetoacetate (*p* = 0.003) but increased levels of Acetone (*p* = 0.001) and Succinate (*p* = 0.036) post IMT.

**Figure 2:**
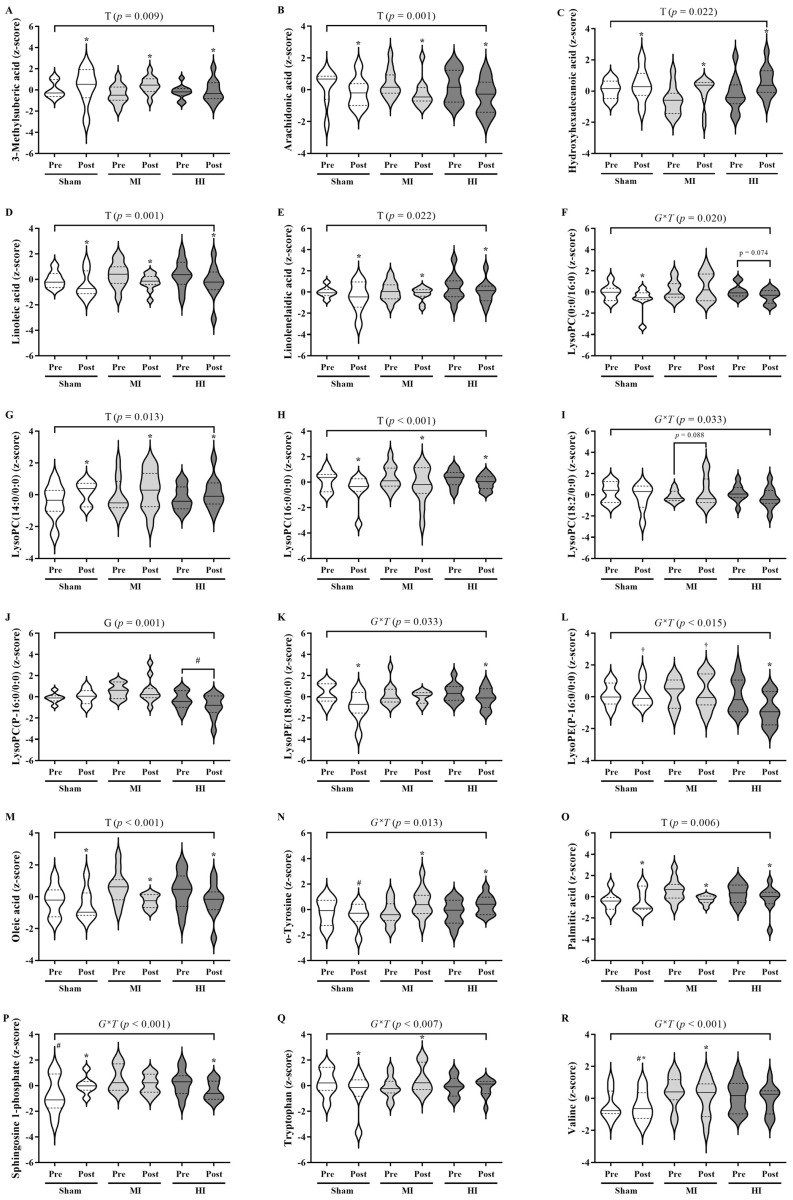
Violin plots for comparisons between and within groups of the annotated metabolites from the UHPLC-HRMS/MS-based metabolomics ((**A**): 3-Methylsuberic acid, (**B**): Arachidonic acid, (**C**): Hydroxyhexadecanoic acid, (**D**): Linoleic acid, (**E**): Linolenelaidic acid, (**F**): LysoPC (0:0/16:0), (**G**): LysoPC (14:0/0:0), (**H**): LysoPC (16:0/0:0), (**I**): LysoPC (18:2/0:0), (**J**): LysoPC (P-16:0/0:0), (**K**): LysoPE (18:0/0:0), (**L**): LysoPE (P-16:0/0:0), (**M**): Oleic acid, (**N**): o-Tyrosine, (**O**): Palmitic acid, (**P**): Sphingosine 1-phosphate, (**Q**): Tryptophan, and (**R**): Valine). Dashed lines represent the 1st and 3rd quartiles; G: Group main effect; T: time main effect; G*T: group–time interaction; * difference from pre (*p* < 0.05); ^#^ difference from MI group (*p* < 0.05); ^†^ difference from HI group (*p* < 0.05); mixed linear model analyses were conducted using SPSS software version 25.0. The figure was generated using GraphPad Prism 9.

For the UHPLC-HRMS/MS-based metabolomics (Figure 2), all groups showed increased metabolic levels of 3-Methylsuberic acid (*p* = 0.009), Hydroxyhexadecanoic acid (*p* = 0.004), and LysoPC (14:0/0:0) (*p* = 0.013) but decreased levels of Arachidonic acid (*p* = 0.001), Linoleic acid (*p* = 0.001), Linolenelaidic acid (*p* = 0.022), LysoPC (16:0/0:0) (*p* < 0.001), LysoPE (18:0/0:0) (*p* < 0.001), Oleic acid (*p* < 0.001), and Palmitic acid (*p* = 0.006) post IMT. The sham group presented increased levels of Sphingosine 1-phosphate (*p* = 0.001) but decreased levels of LysoPC (0:0/16:0) (*p* = 0.024), Tryptophan (*p* = 0.031), and Valine (*p* = 0.022) post IMT. The MI group showed increased levels of o-Tyrosine (*p* = 0.001), Tryptophan (*p* = 0.020), and Valine (*p* < 0.001) but a trend of decreased levels of Sphingosine 1-phosphate (*p* = 0.055). Finally, the HI group presented increased levels of o-Tyrosine (*p* = 0.019) but decreased levels of LysoPE (P-16:0/0:0) (*p* < 0.001) and Sphingosine 1-phosphate (*p* = 0.020) post IMT. In addition, the HI group also showed higher levels of LysoPC (P-16:0/0:0) compared to the MI group (*p* = 0.001).

### 2.3. Metabolite Set Enrichment Analysis

**Figure 3:**
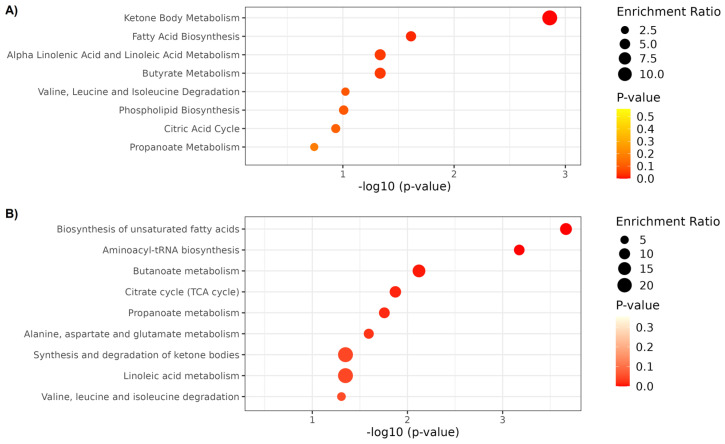
(**A**,**B**). Metabolite set enrichment analysis. The size and color (varying from red to white/yellow) of each circle represent the pathway enrichment ratio (computed by hits/expected hits) and *p*-value, respectively.

**Figure 4:**
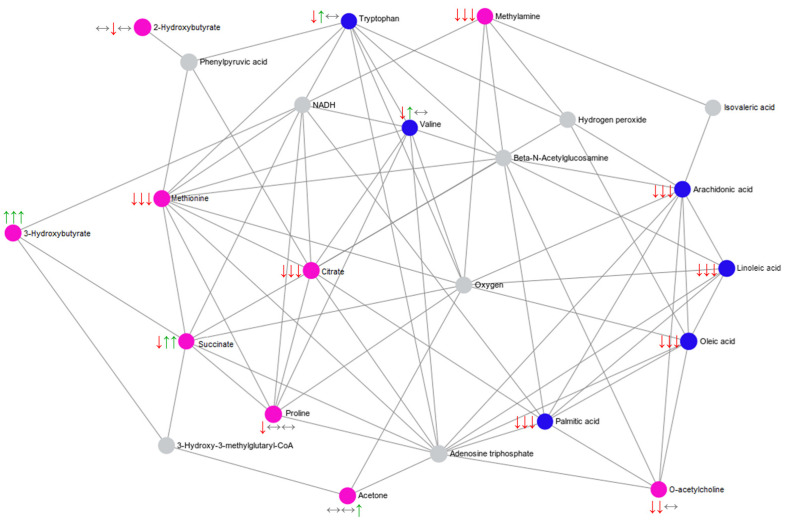
Metabolite–metabolite network analysis. Pink and blue dots indicate metabolites annotated by ^1^H NMR and UHPLC-HRMS/MS, respectively, and the gray dots indicate metabolites that were not annotated but are closely related to other differential metabolites. Green, red and gray arrows indicate upregulation, downregulation, and unchanged metabolites, respectively, after inspiratory muscle training. The first, second and third arrows refer to the sham, MI, and HI groups, respectively.

For the pathway enrichment analysis, metabolites were significantly altered after IMT based on ^1^H NMR (2-Hydroxybutyrate, 3-Hydroxybutyrate, Acetoacetate, Acetone, Citrate, Methionine, Methylamine, O-Acetylcholine, Proline, Propylene glycol, and Succinate) and UHPLC-HRMS/MS (3-Methylsuberic acid, Arachidonic acid, Hydroxyhexadecanoic acid, Linoleic acid, Linolenelaidic acid, LysoPC (0:0/16:0), LysoPC (14:0/0:0), LysoPC (16:0/0:0), LysoPC (18:2/0:0), LysoPE (18:0/0:0), LysoPE (P-16:0/0:0), Oleic acid, o-Tyrosine, Palmitic acid, Sphingosine 1-phosphate, Tryptophan, Valine, and LysoPC (P-16:0/0:0)) was used. A total of 10 distinct metabolic pathways were identified and significantly enriched after IMT (Figure 3): biosynthesis of unsaturated fatty acids (hits: Arachidonic acid, Linoleic acid, Oleic acid, and o-Tyrosine), aminoacyl-tRNA biosynthesis (hits: Methionine, Proline, Tryptophan, and Valine), ketone body metabolism (hits: Acetoacetate, Acetone, and Succinate), butanoate metabolism (hits: Acetoacetate and Succinate), Citrate cycle (hits: Citrate and Succinate), propanoate metabolism (hits: 2-Hydroxybutyrate and Succinate), fatty acid biosynthesis (hits: 3-Hydroxybutyrate, Acetoacetate, and Succinate), alanine, aspartate and glutamate metabolism (hits: Citrate and Succinate), alpha linolenic acid and linoleic acid metabolism (hits: Arachidonic acid and Linoleic acid), and butyrate metabolism (hits: Acetoacetate and Succinate). The complete list of identified pathways is summarized in detail in Table S5. Additionally, we performed a metabolite–metabolite network analysis of the annotated significant metabolites to identify the main interaction between them (Figure 4, Table S6). The identified metabolite that showed the highest degree of centrality with significantly altered metabolites after IMT was Citrate, while the unannotated metabolite was Adenosine triphosphate.

### 2.4. Summary of the Main Findings

**Figure 5:**
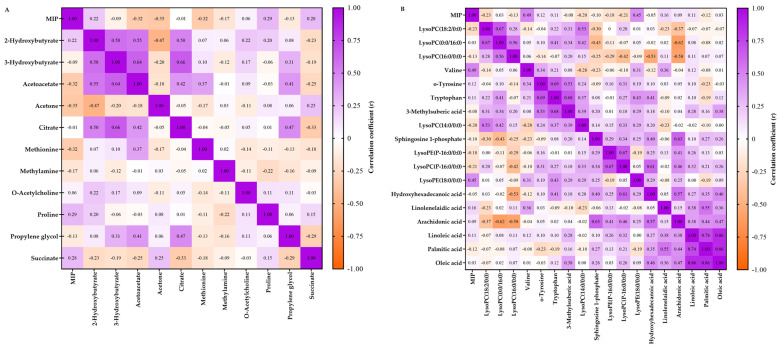
Overall correlation heatmap between changes (%) in metabolic levels and maximal inspiratory pressure after inspiratory muscle training for ^1^H NMR-(**A**) and UHPLC-HRMS/MS-based (**B**) metabolomics. Purple and orange colors represent positive and negative correlations (r), respectively. *p* < 0.05 was assumed for |r| > 0.412 in (**A**) (n = 26) and |r| > 0.374 in (**B**) (n = 28).

A summary of the main findings can be seen in Table 2. Additionally, changes in Valine (r = 0.494, *p* = 0.009) and LysoPE (18:0/0:0) (r = 0.453, *p* = 0.018) levels were positively correlated with gains in MIP after IMT (Figure 5).

## 3. Discussion

This study consisted of a rigorous randomized, double-blind, sham-controlled, parallel-design trial that evaluated the effects of the IMT at different exercise intensities on chronic systemic metabolic adaptations in apparently healthy recreational male cyclists based on ^1^H NMR- and UHPLC-HRMS/MS-based metabolomic approaches. The key findings can be summarized as follows: (I) regardless of the exercise intensity, after 11 weeks of IMT, there were increased serum levels of 3-Hydroxybutyrate, Propylene glycol, 3-Methylsuberic acid, Hydroxyhexadecanoic acid, and LysoPC (14:0/0:0) but decreased levels of Citrate, Methylamine, Arachidonic acid, Linoleic acid, Linolenelaidic acid, LysoPC (16:0/0:0), LysoPE (18:0/0:0), Oleic acid, and Palmitic acid; (II) both the MI and HI groups presented increased levels of Succinate and o-Tyrosine but reduced levels of Acetoacetate and Sphingosine 1-phosphate compared to the sham group; (III) additional alterations after 11 weeks of IMT were observed for the MI group, indicating increased IMS, Tryptophan and Valine levels but decreased 2-Hydroxybutyrate levels compared to the other two groups; (IV) these sets of metabolites mainly indicated the upregulation of ketone body metabolism, fatty acid biosynthesis, and aminoacyl-tRNA biosynthesis, as well as the downregulation of alpha linolenic acid and linoleic acid metabolism and biosynthesis of unsaturated fatty acids.

Interestingly, IMT promoted several changes in lipid metabolism, evidenced by decreased levels of serum fatty acids. Serum fatty acids are derived almost entirely from adipose tissue and play an important role in providing a source of energy for muscle, particularly in the fasting state, its reduced levels likely being attributed to the increased demand for energy of the respiratory muscle after IMT [26]. Oleic acid (omega-9) and Linoleic acid (omega-6) are unsaturated fatty acids, Palmitic acid is a saturated fatty acid, and Arachidonic acid is a polyunsaturated fatty acid. High serum levels of Oleic acid, Linoleic acid, and Palmitic acid have been associated with impairment of the active transepithelial sodium transport mechanisms in the lung [27], pulmonary edema [28], and acute respiratory distress syndrome [27] as well as low serum levels with increased aerobic capacity. On the other hand, Arachidonic acid is responsible for H_2_O_2_ formation and accumulation of reactive oxygen species, which mediates both the development of respiratory muscle dysfunction secondary to infection and dysfunction resulting from an increase in respiratory workload [29]. Changes in arachidonic acid metabolism are evidenced through the increased formation of pro-inflammatory eicosanoids. These are often linked to conditions characterized by low-grade systemic inflammation, such as type 2 diabetes mellitus, hypertension, endothelial dysfunction, and the aging process [30]. Therefore, the reduced levels of Oleic acid, Linoleic acid, Palmitic acid, and Arachidonic acid, which were accompanied by improvement in the maximal inspiratory pressure, reflect the strength of the inspiratory muscles, suggesting, in the present study, the positive metabolic effects of IMT.

Other lipids were also altered by IMT. We observed an increase in serum levels of LysoPC (14:0/0:0), a glycerophospholipid that plays a number of protective or anti-inflammatory effects [31] as a homeostatic regulator, participating in all phases of vascular inflammation by influencing vascular reactivity, endothelial activation and infiltration, and the activation of immune cells [32]. Nevertheless, IMT decreased the serum levels of saturated lysophospholipid [LysoPC (16:0/0:0)] and lysophosphatidylethanolamine [LysoPE (18:0/0:0)]. Studies have shown that the serum elevation of these saturated fatty acids is associated with low-grade inflammation as a result of the aging process and metabolic disorders like insulin resistance, type 2 diabetes, and stroke [33,34,35]. In addition, Sphingosine 1-phosphate also decreased after IMT. This bioactive sphingolipid mediates several cellular processes such as migration, proliferation, inflammation, angiogenesis, morphogenesis, mitochondrial function, and endothelial barrier integrity [36,37]. Elevated Sphingosine 1-phosphate levels have been associated with poor lung function and respiratory disease [37,38,39]. To our knowledge, the relationship between these lipid species and IMT has never been reported. However, based on our findings, the metabolic changes induced by IMT are indicative of improved strength of the inspiratory muscles.

Considering the serum ketone bodies, IMT promoted an increase in serum levels of 3-Hydroxybutyrate and Acetone but a decrease in Acetoacetate levels. 3-Hydroxybutyrate and Acetoacetate are produced in hepatic mitochondria in conditions of reduced carbohydrate availability (i.e., during fasting and starvation) and serve as an alternative fuel source for peripheral tissues including skeletal muscle [40,41]. The increased 3-Hydroxybutyrate levels may result from endogenous production, i.e., elevated ketogenesis derived from free fatty acids [40]. In ketogenesis, most acetoacetate is expected to be reduced to 3-hydroxybutyrate and partly spontaneously decarboxylated to acetone before entering the circulation, which corroborates our findings since decreased serum levels of fatty acids were also observed [40,41,42]. Furthermore, the MI group showed a higher reduction in the serum levels of 2-Hydroxybutyrate compared to the sham and HI groups. The relationship between reduced levels of serum 2-Hydroxybutyrate and IMT is still not well understood; however, lower levels of this metabolite have been associated with improvements in insulin sensitivity [43].

Simultaneously, alterations to amino acid metabolism were observed after IMT, mainly for essential amino acids involved in the translational process through the aminoacyl-tRNA biosynthesis pathway. Although the effects of IMT on this pathway have not been shown in the literature, previous studies demonstrated a positive relationship between the regulation of aminoacyl-tRNA biosynthesis pathway and gains in cardiorespiratory fitness after endurance training programs [44,45]. It is worth pointing out that only the MI group showed an increase in serum levels of Valine and Tryptophan. Valine is a branched-chain amino acid (BCAA), which is required for protein synthesis and maintenance of skeletal muscle [46]. Tryptophan is a glucogenic and ketogenic essential amino acid, a precursor for the important neurotransmitters serotonin and melatonin that regulates the circadian rhythm and plays a critical role in regulating skeletal muscle mass [47,48,49]. Considering that elevated serum levels of Valine and Tryptophan were accompanied by reduced serum fatty acid levels, it could be suggested that individuals from the MI group may benefit from a more efficient mechanism for fatty acid oxidation and muscle protein synthesis after IMT [45,50].

IMT also promoted changes in intermediate metabolites from the Citrate cycle, such as Citrate and Succinate. Citrate can be transported out of the mitochondria and into the cytoplasm, then undergoing breakdown into acetyl-CoA for fatty acid synthesis and oxaloacetate [51]. Taking this into account, it is likely that the reduced serum Citrate levels in our study may be related to the observed decrease in the serum fatty acid levels. Previous studies showed that reduced serum Citrate levels are associated with a lower mortality rate in acute heart failure patients [52] and higher pulmonary function [53], corroborating our findings. Nonetheless, serum levels of Succinate increased after IMT. Succinate serves as a local and/or systemic autacoid, regulating diverse physiological functions and pathological processes [54]. The elevated Succinate serum levels are likely a result of increased flux in the citric acid cycle, brought about by a higher supply of Acetyl-CoA due to fatty acid oxidation, which is reflected in excessive accumulation of its intermediates in the skeletal muscle or liver and consequent diffusion into the circulation [55,56].

Finally, some strengths and limitations should be further discussed in the present study. Even though the study was sufficiently designed to detect significant main effects and interactions of IMT on the serum metabolic profile, it was based on a small number of apparently healthy recreational male cyclists. Therefore, extrapolation of our findings to other populations should be performed with caution. On the other hand, the current study is comparable in sample size to previous studies related to this topic [18,19,20]. This study was also based on a robust, randomized, double-blind, sham-controlled, parallel design trial, with a monitored diet 30 days before the study and until the end of the experiment, and with blood collection obtained after 12 h of fasting. Furthermore, this study presented a comprehensive view of the metabolic adaptations promoted by IMT using a metabolomic approach based on two orthogonal analytical techniques, ^1^H NMR and UHPLC-HRMS/MS. As a result, this approach allowed a powerful integrated platform to maximize the metabolome coverage for different classes of compounds, facilitating the metabolite annotation and pathway enrichment analysis to build more robust models through the integration of multiple data sets. We also recognize the limited number of studies in the literature on metabolomic adaptations related to IMT, which makes discussing our findings challenging.

## 4. Materials and Methods

The data presented in this study were obtained from blood samples of apparently healthy recreational male cyclists who completed an inspiratory muscle training program as part of our previous study [20].

### 4.1. Participants 

A total of twenty-eight apparently healthy recreational male cyclists (20 to 40 years old) were considered for analysis in the study. Information about the recruitment of participants has been previously described [20,57]. Briefly, participants were free from diabetes, hypertension, obesity (body mass index < 30 kg m^−2^), and smoking, and they were regularly engaged in cycling training for 150 min·week^−1^ or more for at least 6 uninterrupted months before the study. Participants were excluded if they presented evidence of abnormalities in the cardiovascular and/or respiratory systems (MIP and maximal expiratory pressure < 60% predicted [58]); used drugs, medications and/or supplements that could interfere with the study protocol and analysis; did not complete 33 total training sessions of 3 days/week^−1^; or changed their physical training and eating habits [18,20]. The study was conducted in accordance with the Declaration of Helsinki, approved by the Human Research Ethics Committee (#2.303.309) at the Universidade Federal de São Carlos (UFSCar) and registered in ClinicalTrials.gov (#NCT02984189, accessed on 21 October 2023). All participants voluntarily signed an informed consent form before they participated in the study.

### 4.2. Experimental Design 

This is a longitudinal, randomized, double-blind, sham-controlled, parallel design trial. The study design was based on the Consolidated Standards of Reporting Trials (CONSORT) recommendations [59]. The full description of the study protocol has been previously described [57].

The procedures of the experimental protocol and IMT were performed at the Cardiovascular Physical Therapy Laboratory—Physical Exercise Research Nucleus at the Department of Physical Therapy—UFSCar. The acquisition of the ^1^H NMR and UHPLC-HRMS/MS metabolomics data as well as processing and chemometrics analysis were conducted at the Laboratory of Nuclear Magnetic Resonance and at the Separare—Chromatography Research Center—Department of Chemistry—UFSCar.

Thirty days before the experimental protocol, all participants underwent a nutritional evaluation and were provided with a dietary plan by a dietitian, which was maintained in entirety during the study to minimize the effects of dietary variations on metabolism during the experimental period. Participants followed the dietary plan based on individual total energy expenditure (estimated) and a balanced diet in terms of macronutrient composition: 45–60% carbohydrate, 10–35% protein and 25–30% fat [60].

Prior to the IMT, blood samples were obtained after a 12 h fasting period. For the physical characterization of the participants at baseline, body composition was measured using dual-energy X-ray absorptiometry (DXA) (Discovery DXA System, Hologic, Marlborough, MA, USA); IMS was measured during maximal inspiratory effort maneuvers using a digital manovacuometer (MVD-300, 179 Globalmed, Porto Alegre, Brazil); and aerobic power ($\dot{V}$O_2peak_) was assessed through a cardiopulmonary exercise test. After completing all baseline tests, participants were randomly assigned into three IMT groups with a 1:1:1 allocation ratio (using opaque envelopes), and 28 participants completed the 11 weeks of IMT and were considered for further analysis: sham (*n* = 7), moderate-intensity group (MI, *n* = 10), and high-intensity groups (HI, *n* = 11). After 11 weeks of IMT, blood samples were reassessed.

For this double-blind study, the participants were blinded to the exact training loads they were submitted, using a barrier over the computer screen. The researchers who performed the assessments did not participate in the training sessions. The researcher who carried out the study analyses (tabulation, data processing, and statistical analyses) did not participate in the randomization or training of the participants. Thus, after tabulating all the data, the same person received from the other researcher the codes for each participant with their respective training group assignment for statistical analysis.

### 4.3. Blood Sample Collection

Venous blood samples were collected at rest, after 12 h of fasting, always in the morning (between 7 and 10 a.m.), by puncture of the antecubital vein in vacuum serum separator tubes (S-Monovette 4.9 mL, Sarstedt, Germany). After 30 min at room temperature, the blood samples were centrifuged at 1450× *g* for 10 min (Sorvall ST Benchtop Centrifuge, Thermo Scientific, Waltham, MA, USA), then serum aliquots were collected and stored at −80 °C until further analysis [61]. All participants were instructed to avoid high-intensity exercises, stimulating foods and drinks (coffee, energy drinks, chocolate, and foods with a lot of sugar), alcoholic beverages, and supplements on the day before blood collection [48].

### 4.4. Respiratory Muscle Strength 

The assessment of respiratory muscle strength was conducted under resting conditions while the participant was seated, using a digital manovacuometer (MVD-300; GlobalMed, Porto Alegre, Brazil) and a nasal clip, adhering to the Brazilian guidelines for measuring maximal static respiratory pressures [62]. All measurements were consistently carried out by the same evaluator. The MIP was determined subsequently to maximal inspiratory efforts (from the residual volume), while the maximal expiratory pressure was assessed after the maximal expiratory effort (from the total lung capacity). Both maneuvers were executed against a tube featuring an occluded distal end, and a 2 mm hole mouthpiece was utilized. The values for maximal respiratory pressures were recorded within the first second following the peak pressure. To obtain accurate and reliable results, a minimum of three maneuvers was performed, with a 30 s interval separating each attempt. The highest reproducible values, with a difference of less than 10%, were extracted from at least three attempts, with the maximal respiratory pressure value being recorded as the highest value. Normal values for this assessment were attributed as proposed for the Brazilian population [63].

### 4.5. Aerobic Power ($\dot{V}$O_2peak_)

The $\dot{V}$O_2peak_ was assessed through a cardiopulmonary exercise test, which was performed on an electromagnetic braking cycle ergometer (CORIVAL V3; Lode BV, Groningen, the Netherlands). After 6 min at rest, the test started with a 3-min free-load warm-up, followed by individualized workload increments (ranging from 25 to 45 W min^−1^) as described by Wasserman et al. [64], with pedaling cadence maintained between 60 and 80 rpm, until the exercise was stopped. The test was followed by 6 min and 1 min of active and passive recovery, respectively [57]. Metabolic and ventilatory measurements were collected, breath by breath, using a gas analyzer (ULTIMA MedGraphics—St Paul, MN/VMAX Encore 29 System—CareFusion, Yorba Linda, CA, USA) and processed in the Breeze Suite 7.1, MedGraphics (St. Paul, MN, USA) software. The highest value of oxygen consumption recorded in the last 30 s of the cardiopulmonary exercise test was assumed as the $\dot{V}$O_2peak_ [65].

### 4.6. Inspiratory Muscle Training

The training protocol details have been described previously [57]. Briefly, the IMT program extended over 11 weeks, 3 days·week^−1^, 1 h per session, and was conducted using a linear inspiratory loading device (PowerBreathe, Ironman K5, HaB Ltd., London, UK). Each session consisted of a 5-min warm-up with a constant loading protocol (50% training load), followed by 3 sets of 15 min of breaths (12 breaths per minute) interspersed with a 1 min interval between sets. Workloads were adjusted every 3 weeks. The IMT intensities used by each group were as follows: 6 cm·H_2_O resistance for the sham group, 60% MIP for the MI, and 85–90% MIP for the HI. The same weekly and total training volume was ensured for each participant over 11 weeks of IMT. Participants were instructed to not change their physical training routine (sham: 6.8 ± 1.8 h·week^−1^ of moderate–vigorous-intensity exercise, MI: 5.1 ± 1.7 h·week^−1^ of moderate–vigorous-intensity exercise, HI: 5.2 ± 1.9 of moderate–vigorous-intensity exercise, *p* = 0.138 for ANOVA test) [66] but to report important changes. No substantive changes were recorded, i.e., none affecting hours of exercise (±1 h·week^−1^).

### 4.7. ^1^H NMR-Based Metabolomics

The sample preparation for the ^1^H NMR-based metabolomics was conducted according previous studies [48,61]. In brief, 3 kDa filters (Amicon Ultra) were washed five times with 500 μL of Milli-Q water, followed by centrifugation at 14,000× *g* for 5 min at 4 °C, and spinning (filter reverse and rotation at 7500× *g* for 60 s) to eliminate residual Milli-Q water. Then, serum samples (500 μL) were filtered by centrifugation at 14,000× *g* for 30 min at 4 °C to promote macromolecule removal. Then, an aliquot of 100 μL of filtered serum was transferred to 5 mm NMR tubes containing 40 μL of 0.1 M phosphate buffer ((monobasic sodium phosphate, NaH_2_PO_4_, 119.97 g∙mol^−1^; dibasic sodium phosphate, Na_2_HPO_4_, 141.96 g∙mol^−1^)), TMSP-d_4_ (3-(trimethylsilyl)-2,2′,3,3′-tetradeuteropropionic acid) at 0.5 mmol⋅l^−1^ as internal reference, and 260 μL of D_2_O (99.9%; Sigma-Aldrich, San Luis, CA, USA).

**Figure 6:**
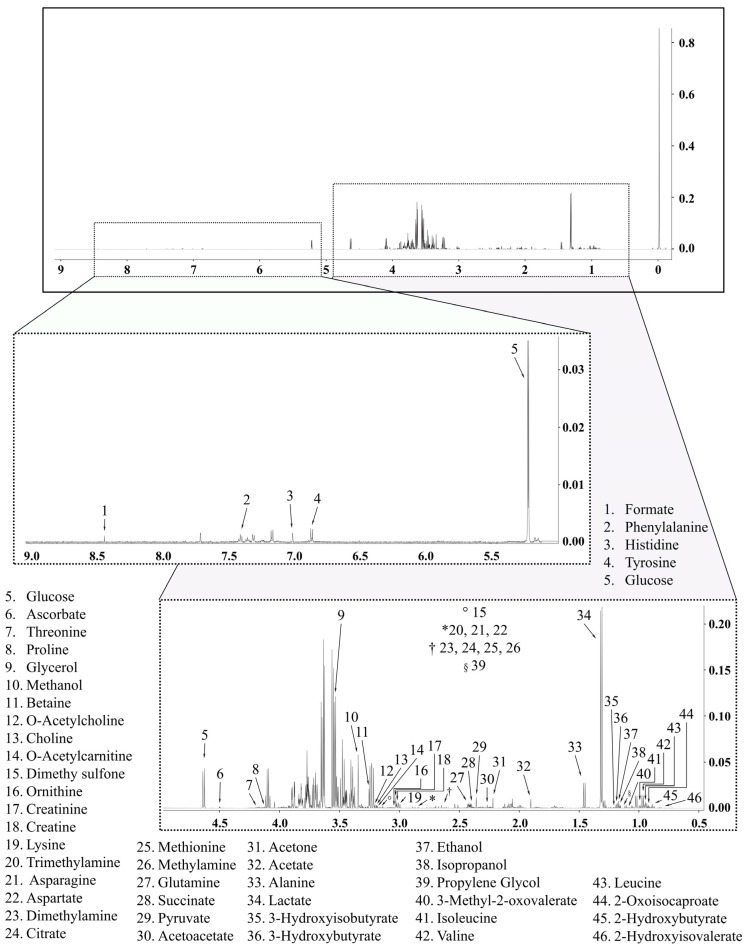
^1^H NMR spectrum with the annotated serum metabolites, 14.1 T, 25 °C in D_2_O. TMSP-d_4_ was used as internal reference.

All NMR experiments were acquired using TopSpin 3.6.2 software in a 14.1 Tesla Bruker spectrometer (600 MHz for hydrogen frequency), equipped with a 5 mm TCI cryoprobe at 298 K. For the ^1^H NMR experiments, a pulse sequence with H_2_O presaturation signal (noesypr1d) was used by adopting a continuous wave, assuming the following acquisition parameters: acquisition time (AQ = 3.63 s), spectral width (SW = 30 ppm), relaxation delay (d1 = 4 s), the 90° pulse time (p1 = 9.5 μs) and number of scans (ns = 128). After spectrum acquisition, baseline corrections, characterization, and quantification of the metabolites detected in the samples (Figure 6) were performed using Suite 8.6 Professional Chenomx software (Chenomx Inc., Edmonton, AB, Canada). All NMR spectra were processed with a line broadening (lb) of 0.3 Hz. The signal of TMSP-d_4_ in ~0.00 ppm was used as an internal reference for metabolite quantification.

Additionally, 2D COrrelated SpectroscopY (COSY), Hetero Single Quantum Coherence (HSQC), and Heteronuclear Multiple Bond Correlation (HMBC) experiments were used to auxiliate in the identification of the most relevant compounds initially annotated by Chenomx software. The assumed parameters for 2D experiments were as follows: SWF1 = 10 ppm and SWF2 = 10 ppm, d1 = 2 s, size of fid in F1 = 256 and F2 = 4096, and ns = 32 for COSY experiment; SWF1 238.82 ppm and SWF2 10 ppm, d1 = 2 s, size of fid in F1 = 256 and F2 = 4096 for both HSQC and HMBC experiments, ns = 128 and ns = 256 for HSQC and HMBC experiments, respectively.

### 4.8. UHPLC-HRMS-Based Metabolomics

The sample preparation for the UHPLC-HRMS/MS-based metabolomics was carried out according to previous studies [48,61]. Serum samples, previously stored at −80 °C, were thawed on ice and vortexed for 15 s. Afterwards, 450 μL of cold methanol was added to 150 μL of serum sample in a microtube to induce protein precipitation and metabolite extraction. The mixture was stored at −20 °C for 5 min and then vortexed for 20 s and centrifuged at 7267× *g* at 4 °C for 10 min. Next, aliquots of 200 μL of the supernatant were transferred to new microtubes and 20 μL of internal standard (5 mmol∙L^−1^ of anhydrous L-Leucine-enkephalin acetate) was added to the samples, which were stored at −20 °C until further analysis. In addition, a blank sample was prepared with 150 μL of water instead of serum, and a quality control (QC) sample was produced by mixing 15 μL of each of the supernatant serum samples that were previously prepared. The blank sample was injected in triplicate at the beginning of the run and the QC sample was injected in triplicate every ten experimental samples. The UHPLC Agilent system (model 1290 Infinity II, Agilent Technologies, Santa Clara, CA, USA) consisted of a binary pump (G712A), a refrigerated autosampler kept at 15 °C (G7129C), and a column compartment (G7129B). HyStar workstation software (HyStar v2, Bruker Daltonics, Bremen, Germany) was used for data acquisition. Compass Data Analysis (DataAnalysis v3.2, Bruker Daltonics) was used for data analysis and processing. The chromatographic analyses were performed with a Zorbax Eclipse XDB-C18 column (100 × 3.0 mm i.d; 3.5 μm) (Agilent Technologies) employing gradient elution using water + 0.1% formic acid (solvent A) and acetonitrile + 0.1% formic acid (solvent B) as mobile phase at a flow rate of 0.4 mL∙min^−1^, and the column temperature was maintained at 40 °C. The total run time was 30 min using the following multistep gradient: 0 min, 1% B; 0–3.0 min, 1–2% B; 3–10 min, 2–30% B; 10–15 min, 30–50% B; 15–18 min, 50–80% B; 18–20 min, 80–90% B; 20–22 min, 90–95% B; 22–26 min, 95–99% B; 26.01–28 min, 99% B, for column cleaning and a conditioning cycle time of 2 min with the same initial conditions of 1% B. The injection volume was 5 μL. Compound detection was performed on a quadrupole time-of-flight mass spectrometer (QqTOF), model Impact HD (Bruker Daltonics) equipped with an electrospray (ESI) interface operating in negative or positive ionization mode. Centroid acquisition mode was used for data collection and storage. The full MS and MS/MS data were acquired through Compass QtofControl v3.4 (Bruker Daltonics) and the data were processed using DataAnalysis 4.2 software (Bruker Daltonics). The optimal ion source parameters were set as follows: capillary voltage, 3600 V and 3000 V for the positive and negative ionization mode, respectively. All other parameters were the same for both ionization modes used: end plate offset, 450 V; nebulizer, 4 bar; dry heater temperature, 180 °C; dry gas flow, 8 l∙min^−1^; quadrupole ion energy, 5 eV, and full-MS scan range, *m*/*z* 50–1300. Dynamic stepping was used for data-dependent acquisition (DDA) MS/MS mode where the collision RF was set to vary between 200.0 and 550.0% Vpp; the transfer time was set to vary 50.0 to 90.0 μs, with 50.0% timing each. The collision energy for the ion fragmentation was programmed to vary from 100 to 250.0% from 20 eV initially set, with the following isolation mass: *m*/*z* 100, 200, and 300: 4 width; for *m*/*z* 700 and 1000: 6 width. Funnels RF 1 and 2 were 250.0 and 150.0 Vpp, respectively. The hexapole RF was 50.0 Vpp, and the quadrupole ion energy was 5.0 eV with a pre-pulse storage of 6.0 μs. The quadrupole ion energy and collision cell energy were both set at 5 eV. The parameters used to trigger the MS/MS fragmentation were 2.0 Hz for low counts (10,000 cts/per 1000 sum) and 4.0 Hz for high counts (100,000 counts/per 1000 sum), using a total cycle time range of 3 s; absolute threshold of 1491 counts (302 counts/per 1000 sum), active exclusion 1 spectra; release after 0.90 min, while the full MS acquisition was set at 2.0 Hz. Internal mass spectrometer calibration was performed with 1 mmol∙L^−1^ of sodium formate prepared in acetonitrile, using a quadratic high-precision calibration (HPC) regression mode. The calibration solution was injected at the end of each analytical run, and all spectra were recalibrated before compound annotation. Bruker Profile Analysis v2.1 software (Bruker Daltonics) was used to process the UHPLC-HRMS/MS data.

Bucket generation was performed with the following parameters: S/N threshold = 2; correlation coefficient threshold = 0.2; minimum compound length = 10 spectra; smoothing width = 1. All features detected by the UHPLC-HRMS/MS were subjected to data processing consisting of the inclusion of features based on values greater than 5% from blank samples, a coefficient of variation (CV%) of QCs samples (mean of replicates) lower than 20%, and the quantity of missing data being lower than 10% in experimental samples [48]. The remaining features were normalized by non-linear local regression (LOESS) to account for the instrumental stability using the Noreva 2.0 software (Figure S1) [67].

Data Analysis v4.2 (Bruker Daltonics) was used for the identification of the fragment ions (MS/MS) of those detected compounds, which were further putatively annotated by comparing their exact mass (error ≤ 10 ppm) and fragment ions with those data in the HMDB (https://hmdb.ca, accessed on 21 October 2023), Mass Bank (https://massbank.eu/MassBank/, accessed on 21 October 2023), CEU Mass Mediator (http://ceumass.eps.uspceu.es/, accessed on 21 October 2023) databases. The annotated compounds were identified based on protonated, deprotonated, and adduct ions for the positive and negative ionization modes (Table S4).

### 4.9. Statistical Analysis

For all variables, the assumption of normality of data distribution and homogeneity of variances were checked using the Shapiro–Wilk test and Levene test, respectively. To compare participant characteristics at baseline, one-way analysis of variance (ANOVA) was used. For the metabolomics data, all variables were Box-Cox transformed [1] and standardized to mean = 0 and multiples of 1 standard deviation (Z-score) [68] to individual feature standardization. To compare the metabolite levels between and within groups, linear mixed models (LMM) estimated using the maximum likelihood method with a Toeplitz covariance matrix structure were used, assuming group (sham, MI and HI groups), time (pre and post IMT), and interaction (group–time) as fixed factors and participants as a random factor. In addition, time was assumed as a repeated-measure effect. Whenever a significant F value was obtained in the ANOVA or LMM, a false discovery rate of 0.2 was applied [69] to account for multiple tests while allowing for hypothesis generation [48] followed by Sidak post hoc adjustment for the purpose of pairwise multiple comparisons. The significance level was set at 5% (*p* < 0.05).

After, to identify meaningful metabolic pathways influenced by IMT, all metabolites that showed significant main effects and interactions in LMM were listed for a metabolite set enrichment analysis (MSEA) based on established human metabolic pathways from the Small Molecule Pathway Database (SMPD) library. This analysis employed an algorithm for over-representation analysis and hypergeometric testing to assess whether specific metabolite sets were represented more than expected by chance within the given compound list [70]. Furthermore, the interconnected network of the most enriched pathways and metabolite–metabolite networks were presented. In addition, the same metabolites listed for MSEA were analyzed using the Pearson correlation coefficient to explore the associations between changes (%) in metabolic levels and changes in MIP gains after IMT.

Univariate analysis was conducted using PASW statistics software version 25.0 (SPSS, Chicago, IL, USA), pathway analyses were performed using the MetaboAnalyst 5.0 webtool, and figures were elaborated using GraphPad Prism 9 or MetaboAnalyst 5.0.

## 5. Conclusions

The IMT promoted significant changes in the serum metabolomic profiles of apparently healthy recreational male cyclists, accompanied by improved strength of the inspiratory muscles. The identified metabolites suggest an increase in the oxidative metabolic processes after IMT at different intensities, with additional evidence for the upregulation of essential amino acid metabolism in the MI group. Although our results highlighted the impact of IMT on serum metabolic adaptations, the underlying mechanisms linking these outcomes remain largely unexplored. Future research should delve into the metabolic and molecular profiles of respiratory muscles and their integration with other tissues and body fluids, and should also include athletes from other sports modalities and even more vulnerable populations, offering potential therapeutic targets to improve performance or monitor respiratory health conditions.

## Figures and Tables

**Table 1:** Participant baseline characteristics. Data are mean ± standard deviation.

Variable	Sham (*n* = 7)			MI (*n* = 11)			HI (*n* = 10)			*p*-Value
Age (years)	28.3	±	5.3	32.8	±	6.4	30.4	±	7.4	0.384
Height (m)	1.79	±	0.04	1.77	±	0.06	1.76	±	0.06	0.481
Body mass (kg)	76.9	±	10.4	75.4	±	8.3	78.6	±	10.9	0.781
BMI (kg·m^−2^)	24.0	±	3.8	24.1	±	1.9	25.3	±	3.7	0.630
Body fat (%)	21.9	±	6.0	20.8	±	3.3	22.6	±	4.6	0.680
MIP (cm·H_2_O)	149.9	±	14.1	159.4	±	24.8	146.8	±	12.8	0.307
$\dot{V}$O_2_ peak (ml·kg^−1^·min^−1^)	41.4	±	3.4	50.7	±	9.4	50.2	±	13.1	0.140

BMI: body mass index; MIP: maximal inspiratory pressure; $\dot{V}$O_2_ peak: aerobic power; MI: moderate-intensity group; HI: high-intensity group.

**Table 2:** Summary of significantly altered metabolites after IMT and their involvement in the most enriched metabolic pathways.

Compound	LMM Effects	Metabolomic Platform	Group			Enriched Pathways
			Sham	MI	HI	
2-Hydroxybutyrate	Interaction	^1^H NMR	↔	↓	↔	Propanoate metabolism
3-Hydroxybutyrate	Interaction	^1^H NMR	↑	↑	↑	Fatty acid biosynthesis
Acetoacetate	Interaction	^1^H NMR	↔	↓	↓	Ketone body metabolism; Butanoate metabolism; Butyrate metabolism; Fatty acid biosynthesis.
Acetone	Interaction	^1^H NMR	↔	↔	↑	Ketone body metabolism
Citrate	Interaction	^1^H NMR	↓	↓	↓	Alanine, aspartate and glutamate metabolism; Citrate cycle
Methionine	Interaction	^1^H NMR	↑	↔	↔	Aminoacyl-tRNA biosynthesis
Methylamine	Interaction	^1^H NMR	↓	↓	↓	
O-Acetylcholine	Interaction	^1^H NMR	↓	↓	↔	
Proline	Interaction	^1^H NMR	↓	↔	↔	Aminoacyl-tRNA biosynthesis
Propylene glycol	Interaction	^1^H NMR	↑	↑	↑	
Succinate	Interaction	^1^H NMR	↓	↑ ^#^	↑	Ketone body metabolism; Butanoate metabolism; Butyrate metabolism; Fatty acid biosynthesis; Propanoate metabolism; Citrate cycle; Alanine, aspartate and glutamate metabolism.
3-Methylsuberic acid	Time	UHPL-HRMS/MS	↑	↑	↑	
Arachidonic acid	Time	UHPL-HRMS/MS	↓	↓	↓	Biosynthesis of unsaturated fatty acids; Alpha linolenic acid and linoleic acid metabolism
Hydroxyhexadecanoic acid	Time	UHPL-HRMS/MS	↑	↑	↑	
Linoleic acid	Time	UHPL-HRMS/MS	↓	↓	↓	Biosynthesis of unsaturated fatty acids; Alpha linolenic acid and linoleic acid metabolism
Linolenelaidic acid	Time	UHPL-HRMS/MS	↓	↓	↓	
LysoPC (0:0/16:0)	Interaction	UHPL-HRMS/MS	↓	↔	↔	
LysoPC (14:0/0:0)	Time	UHPL-HRMS/MS	↑	↑	↑	
LysoPC (16:0/0:0)	Time	UHPL-HRMS/MS	↓	↓	↓	
LysoPC (18:2/0:0)	Interaction	UHPL-HRMS/MS	↔	↔	↔	
LysoPE (18:0/0:0)	Time	UHPL-HRMS/MS	↓	↓	↓	
LysoPE (P-16:0/0:0)	Interaction	UHPL-HRMS/MS	↔	↔	↓	
LysoPC (P-16:0/0:0)	Group	UHPL-HRMS/MS	↔	↔	↓	
Oleic acid	Time	UHPL-HRMS/MS	↓	↓	↓	Biosynthesis of unsaturated fatty acids
o-Tyrosine	Interaction	UHPL-HRMS/MS	↔	↑	↑	Biosynthesis of unsaturated fatty acids
Palmitic acid	Time	UHPL-HRMS/MS	↓	↓	↓	
Sphingosine 1-phosphate	Interaction	UHPL-HRMS/MS	↑	↓ ^‡^	↓	
Tryptophan	Interaction	UHPL-HRMS/MS	↓	↑	↔	Aminoacyl-tRNA biosynthesis
Valine	Interaction	UHPL-HRMS/MS	↓	↑	↔	Aminoacyl-tRNA biosynthesis

IMT: Inspiratory muscular training; LMM: linear mixed model; ↑: upregulation after IMT; ↓ downregulation after IMT; ↔: not changed after IMT; MI: moderate-intensity group; HI: high-intensity group; ^#^ *p* = 0.061; ^‡^ *p* = 0.055.

## Data Availability

All data used to support the findings of this study are included within the article/Supplementary Materials, and they are available upon request from the corresponding author.

## Supplementary Materials

The following supporting information can be downloaded at: https://www.mdpi.com/article/10.3390/ijms242316764/s1.
