# Supplementary material for: Insights into the Serum Metabolic Adaptations in Response to Inspiratory Muscle Training: A Metabolomic Approach Based on 1H NMR and UHPLC-HRMS/MS
Source: Int J Mol Sci. 2023 Nov 25;24(23):16764. doi: 10.3390/ijms242316764 (PMC10706640; doi:10.3390/ijms242316764)
Supplement: Supplementary file 1 [file ijms-24-16764-s001.zip › ijms-2702107-supplementary.pdf]

International Journal of *Molecular Sciences*

Insights into the Serum Metabolic Adaptations in Response to Inspiratory muscle training: A Metabolomics Approach Based on ^{1}H-NMR and UHPLC-HRMS/MS

Alex Castro [1,2,*], Aparecida M. Catai [3], Patrícia Rehder-Santos [3], Étore F. Signini [3], Raphael Martins de Abreu [3,†,‡], Claudio Donisete Da Silva [3], Carla Cristina Dato [4], Regina V. Oliveira [1] and Antônio G. Ferreira [1,*]

[1] Department of Chemistry, Universidade Federal de São Carlos (UFSCar), São Carlos 13565-905, Brazil; oliveirarv@ufscar.br
[2] Biosciences National Laboratory (LNBio), Brazilian Center for Research in Energy and Materials (CNPEM), Campinas 13083-100, Brazil
[3] Department of Physiotherapy, Universidade Federal de São Carlos (UFSCar), Sao Carlos 13565-905, Brazil; mcatai@ufscar.br (A.M.C.); rehderpaty@hotmail.com (P.R.-S.); efsignini@estudante.ufscar.br (É.F.S.); raphaelmartins.abreu@yahoo.com.br (R.M.d.A.); claudio_acrobatica@hotmail.com (C.D.D.S.)
[4] Nutrition Course, Central Paulista University Center, Sao Carlos 13563-470, Brazil; carladatonutricionista@gmail.com
† Current affiliation: Department of Physiotherapy, LUNEX University—International University of Health, Exercise and Sports S.A., 4671 Differdange, Luxembourg.
‡ Current affiliation: LUNEX ASBL Luxembourg Health and Sport Sciences Research Institute, 4671 Differdange, Luxembourg.

[*] Correspondence: ax.castro@yahoo.com.br; alex.castro@lnbio.cnpem.br (A.C.); giba@ufscar.br (A.G.F.)

Supplementary Material

International Journal of *Molecular Sciences*

MDPI

Table S1 - Serum metabolites annotated by ^{1}H NMR spectroscopy and concentration values (mM) for the Sham, MI, and HI groups Pre and Post-inspiratory muscle training. Data are mean ± standard deviation.

**Compound**	**Sham (*n* = 7)**		**MI (*n* = 10)**		**HI (*n* = 10)**		**p and q-values for LMM effects**					
							**G**		**T**		***G*T***	
	**Pre**	**Post**	**Pre**	**Post**	**Pre**	**Post**	***p***	***q***	***p***	***q***	***p***	***q***
2-Hydroxybutyrate	0.031 ± 0.010	0.033 ± 0.014	0.043 ± 0.009	0.032 ± 0.008	0.045 ± 0.024	0.040 ± 0.012	0.080	0.013	**0.027**	0.035	0.038	0.035
2-Hydroxyisovalerate	0.012 ± 0.007	0.010 ± 0.003	0.015 ± 0.012	0.015 ± 0.016	0.011 ± 0.004	0.010 ± 0.002	0.256	0.052	0.313	0.117	0.842	0.174
2-Oxoisocaproate	0.007 ± 0.007	0.005 ± 0.002	0.004 ± 0.002	0.005 ± 0.004	0.005 ± 0.003	0.006 ± 0.002	0.248	0.043	0.705	0.178	0.112	0.043
3-Hydroxybutyrate	0.062 ± 0.100	0.022 ± 0.014	0.102 ± 0.125	0.025 ± 0.008	0.074 ± 0.086	0.036 ± 0.037	0.496	0.126	**0.001**	0.013	0.653	0.126
3-Hydroxyisobutyrate	0.017 ± 0.007	0.017 ± 0.007	0.017 ± 0.004	0.016 ± 0.004	0.021 ± 0.009	0.017 ± 0.006	0.853	0.174	0.286	0.100	0.676	0.130
3-Methyl-2-oxovalerate	0.009 ± 0.003	0.008 ± 0.002	0.008 ± 0.002	0.007 ± 0.001	0.008 ± 0.002	0.007 ± 0.002	0.375	0.100	0.043	0.039	0.861	0.187
Acetate	0.065 ± 0.025	0.056 ± 0.021	0.057 ± 0.017	0.060 ± 0.014	0.046 ± 0.015	0.046 ± 0.017	0.013	0.004	0.681	0.174	0.245	0.083
Acetoacetate	0.034 ± 0.050	0.028 ± 0.029	0.053 ± 0.049	0.019 ± 0.004	0.053 ± 0.047	0.031 ± 0.037	0.439	0.113	**0.001**	0.017	0.029	0.026
Acetone	0.184 ± 0.234	0.119 ± 0.119	0.131 ± 0.119	0.169 ± 0.175	0.238 ± 0.215	0.096 ± 0.113	0.883	0.183	**0.020**	0.030	0.025	0.017
Alanine	0.416 ± 0.123	0.367 ± 0.071	0.338 ± 0.097	0.359 ± 0.094	0.408 ± 0.179	0.390 ± 0.090	0.296	0.065	0.877	0.191	0.285	0.087
Ascorbate	0.042 ± 0.020	0.036 ± 0.021	0.043 ± 0.011	0.037 ± 0.016	0.034 ± 0.013	0.029 ± 0.009	0.311	0.074	0.053	0.048	0.966	0.196
Asparagine	0.045 ± 0.019	0.039 ± 0.017	0.032 ± 0.014	0.034 ± 0.018	0.034 ± 0.026	0.033 ± 0.017	0.290	0.061	0.733	0.183	0.502	0.113
Aspartate	0.030 ± 0.021	0.029 ± 0.015	0.023 ± 0.010	0.025 ± 0.008	0.029 ± 0.012	0.033 ± 0.012	0.182	0.026	0.226	0.087	0.698	0.152
Betaine	0.041 ± 0.022	0.046 ± 0.030	0.046 ± 0.017	0.047 ± 0.020	0.046 ± 0.017	0.046 ± 0.016	0.474	0.122	0.629	0.161	0.994	0.200
Choline	0.011 ± 0.006	0.012 ± 0.004	0.012 ± 0.006	0.011 ± 0.004	0.011 ± 0.004	0.011 ± 0.007	0.710	0.165	0.643	0.165	0.343	0.096
Citrate	0.064 ± 0.018	0.053 ± 0.014	0.064 ± 0.009	0.054 ± 0.009	0.066 ± 0.015	0.059 ± 0.013	0.533	0.143	**0.000**	0.009	0.555	0.117
Creatine	0.018 ± 0.013	0.016 ± 0.009	0.016 ± 0.008	0.019 ± 0.012	0.027 ± 0.023	0.025 ± 0.027	0.702	0.161	0.913	0.196	0.945	0.191
Creatinine	0.102 ± 0.051	0.085 ± 0.021	0.088 ± 0.010	0.085 ± 0.010	0.098 ± 0.041	0.086 ± 0.013	0.944	0.191	0.044	0.043	0.708	0.157
Dimethyl sulfone	0.006 ± 0.003	0.005 ± 0.002	0.007 ± 0.003	0.008 ± 0.002	0.007 ± 0.003	0.006 ± 0.003	0.170	0.022	0.618	0.157	0.170	0.052
Dimethylamine	0.004 ± 0.002	0.004 ± 0.003	0.003 ± 0.000	0.002 ± 0.001	0.004 ± 0.003	0.004 ± 0.003	0.407	0.109	0.165	0.074	0.422	0.109
Ethanol	0.104 ± 0.049	0.108 ± 0.043	0.110 ± 0.062	0.090 ± 0.020	0.149 ± 0.197	0.126 ± 0.059	0.497	0.130	0.474	0.139	0.289	0.091
Formate	0.027 ± 0.007	0.025 ± 0.010	0.024 ± 0.004	0.025 ± 0.005	0.021 ± 0.006	0.021 ± 0.005	0.037	0.009	0.610	0.152	0.194	0.057
Glucose	4.449 ± 1.291	3.718 ± 0.702	4.576 ± 1.420	4.073 ± 0.568	5.020 ± 2.290	4.431 ± 0.924	0.231	0.035	0.106	0.065	0.686	0.139
Glutamine	0.493 ± 0.169	0.475 ± 0.089	0.437 ± 0.053	0.403 ± 0.128	0.495 ± 0.149	0.477 ± 0.112	0.249	0.048	0.219	0.083	0.695	0.148
Glycerol	3.090 ± 2.442	2.683 ± 0.523	2.764 ± 0.608	2.685 ± 0.326	2.909 ± 0.960	2.949 ± 0.932	0.355	0.091	0.127	0.070	0.198	0.061

International Journal of *Molecular Sciences*

MDPI

Histidine	0.091 ± 0.026	0.073 ± 0.012	0.081 ± 0.014	0.083 ± 0.013	0.090 ± 0.032	0.083 ± 0.017	0.678	0.152	0.170	0.078	0.218	0.074
Isoleucine	0.072 ± 0.027	0.062 ± 0.012	0.056 ± 0.010	0.058 ± 0.008	0.062 ± 0.016	0.059 ± 0.012	0.266	0.057	0.544	0.148	0.201	0.070
Isopropanol	0.009 ± 0.006	0.010 ± 0.005	0.009 ± 0.006	0.009 ± 0.005	0.010 ± 0.004	0.008 ± 0.003	0.986	0.200	0.384	0.126	0.345	0.100
Lactate	2.792 ± 1.316	2.477 ± 0.342	2.336 ± 0.542	2.408 ± 1.018	2.422 ± 0.873	2.090 ± 0.595	0.440	0.117	0.289	0.104	0.684	0.135
Leucine	0.094 ± 0.040	0.081 ± 0.011	0.084 ± 0.019	0.079 ± 0.018	0.094 ± 0.029	0.094 ± 0.020	0.236	0.039	0.520	0.143	0.805	0.165
Lysine	0.149 ± 0.063	0.138 ± 0.012	0.152 ± 0.023	0.158 ± 0.020	0.161 ± 0.064	0.149 ± 0.029	0.311	0.078	0.976	0.200	0.583	0.122
Methanol	0.413 ± 0.238	0.394 ± 0.176	0.365 ± 0.166	0.372 ± 0.161	0.516 ± 0.332	0.350 ± 0.122	0.726	0.170	0.312	0.109	0.201	0.065
Methionine	0.023 ± 0.004	0.030 ± 0.002	0.026 ± 0.005	0.026 ± 0.007	0.029 ± 0.007	0.028 ± 0.005	0.338	0.087	0.063	0.061	**0.007**	0.013
Methylamine	0.004 ± 0.004	0.002 ± 0.001	0.003 ± 0.001	0.002 ± 0.002	0.004 ± 0.003	0.002 ± 0.002	0.961	0.196	**0.009**	0.022	0.808	0.170
O-Acetylcarnitine	0.006 ± 0.007	0.008 ± 0.006	0.005 ± 0.002	0.005 ± 0.004	0.006 ± 0.003	0.008 ± 0.007	0.498	0.135	0.650	0.170	0.105	0.039
O-Acetylcholine	0.008 ± 0.002	0.005 ± 0.006	0.010 ± 0.004	0.004 ± 0.003	0.008 ± 0.006	0.006 ± 0.003	0.866	0.178	**0.000**	0.004	0.031	0.030
Ornithine	0.036 ± 0.022	0.031 ± 0.029	0.030 ± 0.025	0.020 ± 0.017	0.026 ± 0.015	0.025 ± 0.018	0.516	0.139	0.272	0.096	0.858	0.183
Phenylalanine	0.069 ± 0.019	0.059 ± 0.008	0.063 ± 0.009	0.061 ± 0.009	0.065 ± 0.023	0.062 ± 0.010	0.931	0.187	0.062	0.057	0.237	0.078
Proline	0.174 ± 0.143	0.107 ± 0.050	0.100 ± 0.046	0.123 ± 0.067	0.141 ± 0.052	0.176 ± 0.069	0.107	0.017	0.746	0.187	**0.006**	0.009
Propylene glycol	0.006 ± 0.004	0.004 ± 0.002	0.004 ± 0.002	0.003 ± 0.001	0.004 ± 0.002	0.003 ± 0.001	0.204	0.030	**0.013**	0.026	0.854	0.178
Pyruvate	0.034 ± 0.025	0.048 ± 0.041	0.059 ± 0.025	0.041 ± 0.025	0.060 ± 0.042	0.044 ± 0.015	0.338	0.083	0.229	0.091	0.025	0.022
Succinate	0.009 ± 0.004	0.005 ± 0.002	0.004 ± 0.001	0.006 ± 0.005	0.005 ± 0.002	0.006 ± 0.001	0.365	0.096	0.439	0.135	**0.000**	0.004
Threonine	0.099 ± 0.039	0.101 ± 0.044	0.086 ± 0.043	0.068 ± 0.049	0.101 ± 0.067	0.088 ± 0.051	0.388	0.104	0.330	0.122	0.687	0.143
Trimethylamine	0.002 ± 0.001	0.002 ± 0.001	0.002 ± 0.000	0.002 ± 0.001	0.002 ± 0.002	0.002 ± 0.001	0.700	0.157	0.313	0.113	0.150	0.048
Tyrosine	0.075 ± 0.029	0.065 ± 0.015	0.066 ± 0.009	0.066 ± 0.009	0.076 ± 0.034	0.074 ± 0.019	0.569	0.148	0.398	0.130	0.383	0.104
Valine	0.278 ± 0.113	0.232 ± 0.037	0.221 ± 0.028	0.216 ± 0.047	0.255 ± 0.079	0.239 ± 0.052	0.302	0.070	0.057	0.052	0.759	0.161

Data are mean ± standard deviation. MI: 60% maximal inspiratory pressure (MIP); HI: ~90% MIP; LMM: Linear Mixed Model; p: P-values for the LMM; q: Adjusted p-values for the false discovery rate (FDR); G: Group main effect; T: Time main effect; *G*T*: *Group*Time* interaction. Bold values indicate significant main effects adjusted for the FDR of 0.2 at the significance level of 5%. Data were transformed by Box and Cox procedure before analysis and presented in their original scale. Analyzes were conducted in the SPSS software, version 25.0.

Table S2 - Serum compounds detected by UHPLC-HRMS/MS in positive ionization mode and peak intensities (u.a.) for the Sham, MI, and HI groups Pre and Post-inspiratory muscle training. Data are mean ± standard deviation.

**Compound [retention time (min):mass-to-charge ratio (*m/z*)]**	**Sham (n = 7)**		**MI (n = 11)**		**HI (n = 10)**		**p and q-values for LMM effects**					
							**G**		**T**		***G*T***	
	**Pre**	**Post**	**Pre**	**Post**	**Pre**	**Post**	***p***	***q***	***p***	***q***	***p***	***q***
1.69 : 146.030	1.098 ± 0.324	1.189 ± 0.341	1.231 ± 0.524	1.317 ± 0.295	1.269 ± 0.260	1.333 ± 0.330	0.303	0.095	0.064	0.044	0.681	0.163
1.90 : 120.066	1.053 ± 0.165	0.842 ± 0.204	0.889 ± 0.140	1.029 ± 0.395	0.993 ± 0.332	0.958 ± 0.250	0.965	0.200	0.425	0.117	**0.007**	0.012
1.97 : 162.113	0.915 ± 0.139	0.773 ± 0.386	0.916 ± 0.233	0.934 ± 0.198	1.069 ± 0.202	1.041 ± 0.244	0.163	0.068	0.441	0.120	0.672	0.159
2.07 : 203.054	0.951 ± 0.156	0.839 ± 0.400	0.972 ± 0.220	1.160 ± 0.188	0.985 ± 0.153	1.015 ± 0.172	0.104	0.049	0.053	0.037	**0.008**	0.015
2.09 : 104.077	0.969 ± 0.196	0.814 ± 0.426	0.871 ± 0.300	1.034 ± 0.397	1.009 ± 0.300	0.796 ± 0.346	0.837	0.185	0.461	0.129	0.166	0.088
2.13 : 116.070	0.879 ± 0.229	0.734 ± 0.322	0.741 ± 0.336	0.885 ± 0.548	0.885 ± 0.268	0.947 ± 0.488	0.693	0.163	0.851	0.178	0.284	0.102
2.31 : 169.039	1.035 ± 0.300	0.822 ± 0.387	1.084 ± 0.247	1.112 ± 0.312	1.100 ± 0.232	1.108 ± 0.125	0.218	0.080	0.177	0.071	0.066	0.056
3.05 : 182.087	0.920 ± 0.204	0.823 ± 0.418	1.007 ± 0.119	1.089 ± 0.206	0.971 ± 0.144	1.104 ± 0.192	0.086	0.041	0.090	0.056	0.064	0.054
3.25 : 136.067	0.943 ± 0.276	0.967 ± 0.335	1.097 ± 0.179	1.170 ± 0.286	1.003 ± 0.139	1.138 ± 0.237	0.100	0.044	0.082	0.054	0.502	0.146
3.41 : 86.096	1.074 ± 0.147	0.889 ± 0.409	0.975 ± 0.242	1.102 ± 0.181	0.945 ± 0.207	0.978 ± 0.141	0.410	0.112	0.804	0.173	**0.015**	0.024
3.42 : 132.096	1.061 ± 0.197	0.887 ± 0.422	0.983 ± 0.154	1.117 ± 0.199	0.976 ± 0.138	1.050 ± 0.190	0.775	0.173	0.217	0.078	**0.009**	0.017
3.88 : 153.054	0.946 ± 0.428	1.191 ± 0.563	0.943 ± 0.314	1.004 ± 0.390	0.982 ± 0.423	1.033 ± 0.362	0.904	0.195	0.296	0.090	0.777	0.185
3.90 : 137.046	1.100 ± 0.746	0.824 ± 0.414	0.970 ± 0.455	1.021 ± 0.326	0.995 ± 0.233	0.980 ± 0.256	0.801	0.176	0.251	0.080	0.107	0.080
5.97 : 100.093	0.599 ± 0.714	1.188 ± 1.150	0.898 ± 0.603	0.872 ± 0.584	1.509 ± 1.625	1.258 ± 1.143	0.588	0.149	0.206	0.076	0.080	0.063
6.64 : 120.081	0.963 ± 0.100	0.842 ± 0.406	0.977 ± 0.242	1.117 ± 0.258	0.937 ± 0.148	0.988 ± 0.109	0.171	0.071	0.313	0.098	**0.035**	0.037
6.80 : 166.086	0.975 ± 0.107	0.852 ± 0.396	0.982 ± 0.241	1.109 ± 0.263	0.935 ± 0.156	0.981 ± 0.115	0.205	0.076	0.419	0.115	0.047	0.044
7.22 : 209.101	0.872 ± 0.452	1.102 ± 0.426	0.852 ± 0.288	1.137 ± 0.314	0.886 ± 0.234	0.899 ± 0.174	0.554	0.144	**0.001**	0.005	0.052	0.049
7.47 : 253.126	0.984 ± 0.516	1.155 ± 0.606	0.972 ± 0.369	1.278 ± 0.638	0.887 ± 0.307	1.035 ± 0.559	0.590	0.151	0.031	0.027	0.664	0.156
7.71 : 185.128	0.926 ± 0.173	0.923 ± 0.500	1.153 ± 0.468	0.982 ± 0.294	0.947 ± 0.408	0.765 ± 0.256	0.210	0.078	0.025	0.022	0.886	0.190
7.75 : 472.683	0.956 ± 0.548	0.848 ± 0.315	0.955 ± 0.503	1.029 ± 0.429	0.852 ± 0.445	0.736 ± 0.192	0.432	0.117	0.948	0.190	0.776	0.183
8.09 : 239.148	0.980 ± 0.421	1.002 ± 0.550	0.939 ± 0.244	1.088 ± 0.419	0.870 ± 0.177	0.902 ± 0.367	0.534	0.132	0.795	0.171	0.392	0.122
8.14 : 181.072	1.091 ± 0.772	1.162 ± 1.187	0.920 ± 0.527	1.188 ± 0.914	0.756 ± 0.694	1.198 ± 1.147	0.837	0.188	0.365	0.100	0.462	0.129
8.14 : 205.097	1.074 ± 0.141	0.844 ± 0.388	0.827 ± 0.166	1.173 ± 0.271	0.859 ± 0.156	1.020 ± 0.289	0.685	0.161	0.021	0.017	**0.000**	0.002
8.24 : 247.137	0.474 ± 0.385	0.555 ± 0.216	1.207 ± 1.235	1.101 ± 1.234	0.886 ± 0.707	0.875 ± 0.655	0.120	0.056	0.526	0.139	0.077	0.061
8.70 : 114.089	0.298 ± 0.323	1.694 ± 3.334	0.285 ± 0.203	0.308 ± 0.262	0.468 ± 0.450	0.411 ± 0.305	0.539	0.134	**0.015**	0.015	**0.004**	0.010
8.82 : 155.152	0.867 ± 0.172	1.231 ± 0.574	1.093 ± 0.262	0.992 ± 0.278	0.753 ± 0.104	1.137 ± 0.350	0.450	0.120	**0.005**	0.012	**0.001**	0.005
9.70 : 113.059	0.995 ± 0.202	1.561 ± 1.576	1.004 ± 0.241	1.112 ± 0.371	0.887 ± 0.129	0.949 ± 0.255	0.373	0.105	0.311	0.095	0.979	0.200
10.23 : 134.057	0.995 ± 0.309	1.146 ± 0.366	1.141 ± 0.455	1.104 ± 0.376	0.984 ± 0.647	0.966 ± 0.434	0.546	0.139	0.486	0.137	0.708	0.168

International Journal of *Molecular Sciences*

MDPI

10.44 : 180.067	0.683 ± 0.215	0.719 ± 0.233	1.442 ± 0.823	1.261 ± 0.999	0.797 ± 0.905	0.961 ± 0.822	0.133	0.061	0.564	0.144	0.295	0.107
10.56 : 603.791	0.856 ± 0.550	0.643 ± 0.365	1.034 ± 0.315	0.963 ± 0.522	0.987 ± 0.449	1.153 ± 0.301	0.137	0.063	0.568	0.149	0.090	0.068
10.56 : 510.759	0.887 ± 0.305	0.879 ± 0.428	0.934 ± 0.380	0.891 ± 0.420	0.910 ± 0.335	1.192 ± 0.411	0.451	0.122	0.374	0.107	0.050	0.046
10.87 : 516.300	0.932 ± 0.186	0.925 ± 0.273	0.963 ± 0.222	1.068 ± 0.192	0.952 ± 0.118	0.901 ± 0.207	0.340	0.102	0.862	0.180	0.380	0.120
10.87 : 733.334	0.618 ± 0.407	0.698 ± 0.762	1.126 ± 0.796	1.176 ± 0.909	1.019 ± 0.694	1.236 ± 0.671	0.102	0.046	0.563	0.141	0.545	0.149
11.13 : 604.355	0.908 ± 0.319	1.021 ± 0.166	1.020 ± 0.198	1.050 ± 0.302	1.002 ± 0.185	0.908 ± 0.192	0.577	0.146	0.624	0.156	0.075	0.059
11.36 : 648.378	1.043 ± 0.185	1.050 ± 0.274	0.965 ± 0.295	1.093 ± 0.385	1.084 ± 0.236	0.827 ± 0.139	0.519	0.129	0.156	0.068	**0.002**	0.007
11.36 : 560.323	0.940 ± 0.198	0.870 ± 0.342	0.964 ± 0.200	1.140 ± 0.174	1.016 ± 0.066	1.039 ± 0.153	0.131	0.059	0.037	0.029	**0.009**	0.020
11.62 : 562.340	1.002 ± 0.292	1.181 ± 0.185	0.944 ± 0.135	1.021 ± 0.170	0.962 ± 0.291	0.963 ± 0.197	0.244	0.088	**0.004**	0.010	0.174	0.090
11.76 : 698.786	0.830 ± 0.391	0.790 ± 0.425	1.099 ± 0.436	0.899 ± 0.478	1.007 ± 0.531	1.207 ± 0.463	0.205	0.073	0.667	0.168	**0.039**	0.039
12.21 : 176.072	1.023 ± 0.277	0.824 ± 0.217	0.860 ± 0.398	0.875 ± 0.339	1.047 ± 0.290	1.162 ± 0.588	0.236	0.085	0.567	0.146	0.311	0.112
13.64 : 585.270	1.323 ± 1.041	1.045 ± 0.663	1.250 ± 0.517	1.024 ± 0.423	1.107 ± 0.692	0.962 ± 0.648	0.706	0.168	0.101	0.059	0.933	0.195
13.97 : 183.988	0.957 ± 0.601	1.155 ± 0.599	0.880 ± 0.274	0.913 ± 0.473	0.844 ± 0.289	0.892 ± 0.483	0.810	0.180	0.622	0.154	0.484	0.134
14.97 : 316.247	0.802 ± 0.522	0.705 ± 0.427	1.006 ± 0.517	0.773 ± 0.209	1.145 ± 1.192	1.337 ± 1.587	0.652	0.156	0.887	0.188	0.288	0.105
15.27 : 290.258	0.979 ± 0.212	1.330 ± 1.479	0.993 ± 0.305	1.207 ± 1.249	0.943 ± 0.262	0.917 ± 0.289	0.954	0.198	0.205	0.073	0.716	0.173
15.70 : 187.126	0.684 ± 0.748	0.626 ± 0.570	0.974 ± 0.560	1.193 ± 0.811	0.675 ± 0.456	0.888 ± 0.790	0.060	0.027	0.306	0.093	0.705	0.166
16.22 : 274.274	1.165 ± 0.503	0.804 ± 0.234	0.945 ± 0.310	0.836 ± 0.371	1.212 ± 0.688	0.976 ± 0.317	0.493	0.124	**0.002**	0.007	0.317	0.117
16.82 : 416.309	1.429 ± 0.667	1.300 ± 1.398	1.006 ± 0.625	1.635 ± 1.262	0.956 ± 0.518	0.945 ± 0.727	0.554	0.141	0.958	0.195	0.148	0.085
18.57 : 330.336	1.149 ± 0.593	0.938 ± 0.966	0.993 ± 0.443	0.787 ± 0.881	1.003 ± 0.583	0.905 ± 0.446	0.858	0.190	0.028	0.024	0.500	0.141
18.64 : 468.309	0.755 ± 0.405	0.944 ± 0.558	0.912 ± 0.453	1.109 ± 0.574	0.887 ± 0.386	1.182 ± 0.556	0.431	0.115	0.024	0.020	0.736	0.180
18.80 : 520.340	0.873 ± 0.165	0.764 ± 0.364	0.834 ± 0.139	0.889 ± 0.284	0.877 ± 0.145	0.771 ± 0.226	0.809	0.178	0.148	0.066	0.092	0.076
18.88 : 1039.673	0.865 ± 0.287	0.731 ± 0.389	0.770 ± 0.162	0.903 ± 0.425	0.851 ± 0.238	0.722 ± 0.302	0.865	0.193	0.382	0.110	**0.033**	0.034
19.26 : 400.353	0.784 ± 0.166	0.973 ± 0.339	1.314 ± 0.413	1.316 ± 0.537	1.198 ± 0.379	1.011 ± 0.461	0.025	0.010	0.986	0.198	0.083	0.066
19.28 : 494.332	0.709 ± 0.223	0.612 ± 0.380	1.041 ± 0.781	0.928 ± 0.385	0.941 ± 0.442	0.898 ± 0.315	0.112	0.051	0.271	0.088	0.501	0.144
19.45 : 496.340	0.941 ± 0.109	0.778 ± 0.345	0.974 ± 0.127	1.009 ± 0.179	0.958 ± 0.090	0.889 ± 0.100	0.068	0.029	0.072	0.051	**0.020**	0.029
19.57 : 991.674	0.966 ± 0.158	0.785 ± 0.354	1.020 ± 0.175	0.854 ± 0.354	1.000 ± 0.131	0.939 ± 0.111	0.542	0.137	**0.000**	0.002	0.498	0.139
19.66 : 480.321	0.928 ± 0.208	0.806 ± 0.372	0.989 ± 0.270	0.919 ± 0.398	0.896 ± 0.262	0.813 ± 0.208	0.513	0.127	0.056	0.039	0.905	0.193
19.69 : 522.356	0.906 ± 0.225	0.764 ± 0.378	0.954 ± 0.209	0.934 ± 0.321	0.858 ± 0.189	0.793 ± 0.195	0.266	0.093	0.067	0.049	0.493	0.137
19.72 : 428.373	0.750 ± 0.197	1.099 ± 0.490	1.203 ± 0.439	1.231 ± 0.490	1.071 ± 0.223	0.900 ± 0.313	0.030	0.012	0.647	0.161	0.054	0.051
20.02 : 482.336	0.911 ± 0.193	0.747 ± 0.338	1.097 ± 0.368	1.109 ± 0.576	0.873 ± 0.264	0.764 ± 0.222	0.034	0.017	0.138	0.063	0.445	0.124
22.11 : 524.371	0.905 ± 0.194	0.774 ± 0.348	1.051 ± 0.269	1.098 ± 0.286	0.972 ± 0.170	0.948 ± 0.308	0.048	0.022	0.400	0.112	0.255	0.098
22.46 : 870.561	0.825 ± 0.420	0.623 ± 0.370	0.973 ± 0.386	1.094 ± 0.616	1.095 ± 0.352	0.990 ± 0.321	0.079	0.039	0.365	0.102	0.136	0.083
22.80 : 550.382	0.916 ± 0.286	1.072 ± 0.716	0.949 ± 0.311	1.137 ± 0.493	0.872 ± 0.223	0.726 ± 0.208	0.073	0.032	0.826	0.176	**0.011**	0.022

23.25 : 760.505	0.845 ± 0.313	0.868 ± 0.687	0.928 ± 0.373	1.135 ± 0.943	0.979 ± 0.337	0.919 ± 0.300	0.702	0.166	0.877	0.185	0.723	0.178
23.32 : 826.545	0.809 ± 0.317	0.524 ± 0.347	0.898 ± 0.342	0.949 ± 0.600	0.993 ± 0.337	0.845 ± 0.300	0.141	0.066	0.048	0.034	0.101	0.078
23.32 : 738.492	0.821 ± 0.326	0.830 ± 0.539	0.893 ± 0.340	1.022 ± 0.643	1.001 ± 0.342	0.976 ± 0.336	0.672	0.159	0.661	0.166	0.709	0.171
23.34 : 694.464	0.834 ± 0.237	0.950 ± 0.662	0.920 ± 0.297	0.917 ± 0.514	0.970 ± 0.299	1.018 ± 0.315	0.757	0.171	0.864	0.183	0.831	0.188
23.58 : 256.262	0.723 ± 0.314	0.790 ± 0.409	1.004 ± 0.186	0.907 ± 0.236	0.961 ± 0.209	0.934 ± 0.267	0.073	0.034	0.654	0.163	0.312	0.115
23.75 : 650.441	0.773 ± 0.164	0.695 ± 0.396	0.829 ± 0.260	0.796 ± 0.375	0.881 ± 0.231	0.897 ± 0.221	0.319	0.100	0.486	0.134	0.719	0.176
23.83 : 737.438	0.970 ± 0.074	1.476 ± 1.233	0.977 ± 0.124	1.440 ± 1.644	1.035 ± 0.125	1.122 ± 0.611	0.827	0.183	0.990	0.200	0.180	0.093
24.15 : 666.469	1.101 ± 0.683	1.284 ± 1.111	1.128 ± 0.579	1.060 ± 0.412	1.019 ± 0.276	0.848 ± 0.569	0.385	0.107	0.457	0.124	0.212	0.095
24.23 : 912.627	0.825 ± 0.421	0.752 ± 0.551	0.921 ± 0.434	1.112 ± 1.052	1.064 ± 0.627	1.128 ± 0.664	0.392	0.110	0.602	0.151	0.584	0.151
24.41 : 597.407	0.783 ± 0.576	0.459 ± 0.310	0.871 ± 0.460	1.163 ± 1.082	1.066 ± 0.620	1.102 ± 0.698	0.304	0.098	0.370	0.105	0.460	0.127
24.63 : 553.381	0.791 ± 0.588	0.448 ± 0.365	0.931 ± 0.421	1.183 ± 1.005	1.072 ± 0.661	1.121 ± 0.711	0.057	0.024	0.107	0.061	0.092	0.073
24.66 : 956.641	0.742 ± 0.398	0.923 ± 1.213	0.879 ± 0.392	1.100 ± 1.019	1.058 ± 0.613	1.051 ± 0.634	0.625	0.154	0.955	0.193	0.954	0.198
24.91 : 531.371	0.742 ± 0.580	0.494 ± 0.372	0.965 ± 0.500	1.107 ± 1.004	1.050 ± 0.653	1.215 ± 0.826	0.077	0.037	0.268	0.085	0.304	0.110
25.15 : 1000.672	0.754 ± 0.470	0.423 ± 0.344	0.921 ± 0.428	1.152 ± 1.063	1.044 ± 0.560	1.085 ± 0.566	0.016	0.002	0.041	0.032	**0.018**	0.027
25.35 : 916.648	0.706 ± 0.425	0.427 ± 0.344	0.853 ± 0.403	1.136 ± 1.137	1.036 ± 0.578	0.979 ± 0.537	0.022	0.007	0.057	0.041	**0.039**	0.041
25.48 : 740.550	0.692 ± 0.217	0.732 ± 0.533	0.813 ± 0.382	1.101 ± 1.082	0.973 ± 0.449	1.033 ± 0.431	0.233	0.083	0.466	0.132	0.476	0.132
25.54 : 652.493	0.808 ± 0.338	0.603 ± 0.375	0.958 ± 0.335	1.237 ± 1.158	0.938 ± 0.376	1.104 ± 0.449	0.031	0.015	0.263	0.083	**0.022**	0.032
25.74 : 564.439	0.835 ± 0.373	0.651 ± 0.451	0.973 ± 0.234	1.195 ± 0.887	0.916 ± 0.325	1.024 ± 0.320	0.047	0.020	0.626	0.159	0.284	0.100
25.99 : 476.394	0.806 ± 0.264	0.892 ± 0.308	0.965 ± 0.204	1.151 ± 0.707	0.898 ± 0.285	1.022 ± 0.273	0.259	0.090	0.065	0.046	0.652	0.154
26.44 : 284.295	0.747 ± 0.299	0.724 ± 0.375	0.960 ± 0.229	1.036 ± 0.327	0.979 ± 0.194	0.821 ± 0.267	0.115	0.054	0.452	0.122	0.090	0.071
27.04 : 310.309	0.706 ± 0.308	0.729 ± 0.391	0.975 ± 0.144	0.931 ± 0.332	0.954 ± 0.220	0.868 ± 0.305	0.017	0.005	0.460	0.127	0.680	0.161

MI: 60% maximal inspiratory pressure (MIP); HI: ~90% MIP; LMM: Linear Mixed Model; p: p-values for the LMM; q: Adjusted p-values for the false discovery rate (FDR); G: Group main effect; T: Time main effect; *G*T*: *Group*Time* interaction. Bold values indicate significant main effects adjusted for the FDR of 0.2 at the significance level of 5%. Data were transformed by Box and Cox procedure before analysis and presented in their original scale. Analyzes were conducted in the SPSS software, version 25.0.

International Journal of *Molecular Sciences*

MDPI

Table S3 - Serum compounds detected by UHPLC-HRMS/MS in negative ionization mode and peak intensities (u.a.) for the Sham, MI, and HI groups Pre and Post-inspiratory muscle training. Data are mean ± standard deviation.

**Compound [retention time (min):mass-to-charge ratio (*m/z*)]**	**Sham (n = 7)**		**MI (n = 11)**		**HI (n = 10)**		**p and q-values for LMM effects**					
							**G**		**T**		***G*T***	
	**Pre**	**Post**	**Pre**	**Post**	**Pre**	**Post**	***p***	***q***	***p***	***q***	***p***	***q***
1.07 : 154.062	1.005 ± 0.267	0.984 ± 0.287	1.084 ± 0.196	1.281 ± 0.305	1.267 ± 0.463	0.867 ± 0.338	0.201	0.050	0.324	0.093	0.018	0.022
1.07 : 96.922	0.989 ± 0.121	0.972 ± 0.095	0.996 ± 0.106	1.012 ± 0.098	1.046 ± 0.110	0.990 ± 0.085	0.525	0.123	0.381	0.110	0.172	0.078
1.08 : 94.925	1.002 ± 0.089	0.972 ± 0.099	0.997 ± 0.119	1.028 ± 0.078	1.057 ± 0.088	0.992 ± 0.125	0.339	0.086	0.402	0.116	0.134	0.063
1.09 : 92.928	1.003 ± 0.088	0.984 ± 0.097	1.010 ± 0.103	1.039 ± 0.087	1.076 ± 0.104	1.015 ± 0.122	0.763	0.161	0.835	0.179	0.088	0.054
1.09 : 150.886	1.003 ± 0.101	0.941 ± 0.162	0.976 ± 0.076	0.935 ± 0.073	0.932 ± 0.100	1.010 ± 0.103	0.723	0.153	0.164	0.056	0.010	0.013
1.09 : 152.884	0.996 ± 0.135	0.973 ± 0.135	0.975 ± 0.086	0.971 ± 0.109	0.950 ± 0.103	1.053 ± 0.119	0.823	0.172	0.696	0.159	0.490	0.135
1.13 : 146.046	1.077 ± 0.837	1.112 ± 0.519	0.974 ± 0.324	1.153 ± 0.720	1.048 ± 0.590	0.862 ± 0.436	0.355	0.092	**0.523**	0.133	0.773	0.174
1.14 : 145.062	1.092 ± 0.143	1.064 ± 0.629	0.886 ± 0.296	0.987 ± 0.111	0.955 ± 0.094	1.021 ± 0.120	0.525	0.125	0.678	0.155	0.017	0.020
1.17 : 112.986	0.918 ± 0.284	0.896 ± 0.291	0.926 ± 0.274	0.800 ± 0.236	0.864 ± 0.314	1.085 ± 0.299	0.886	0.189	0.009	0.026	0.023	0.024
1.18 : 195.811	1.047 ± 0.263	0.992 ± 0.334	0.983 ± 0.266	0.970 ± 0.206	1.145 ± 0.263	0.874 ± 0.284	0.033	0.013	0.501	0.129	0.000	0.002
1.18 : 154.919	0.905 ± 0.345	1.143 ± 0.243	1.017 ± 0.249	0.642 ± 0.297	1.063 ± 0.258	1.077 ± 0.190	0.064	0.024	0.662	0.148	0.151	0.072
1.23 : 217.030	0.908 ± 0.134	0.859 ± 0.263	0.978 ± 0.117	1.020 ± 0.134	1.038 ± 0.127	0.988 ± 0.125	0.246	0.061	0.303	0.090	0.814	0.178
1.46 : 119.035	0.798 ± 0.410	0.784 ± 0.405	1.214 ± 0.492	1.058 ± 0.506	1.077 ± 0.483	0.972 ± 0.408	0.664	0.146	0.380	0.108	0.000	0.004
1.46 : 116.072	0.999 ± 0.329	0.785 ± 0.383	0.842 ± 0.217	1.123 ± 0.208	0.938 ± 0.358	0.995 ± 0.275	0.728	0.157	0.517	0.131	0.540	0.137
1.55 : 135.030	0.854 ± 0.470	0.782 ± 0.602	0.852 ± 0.415	0.919 ± 0.374	0.892 ± 0.348	0.681 ± 0.228	0.864	0.181	0.005	0.017	0.184	0.080
1.58 : 96.960	0.890 ± 0.285	1.053 ± 0.225	0.907 ± 0.211	0.956 ± 0.278	0.856 ± 0.133	0.962 ± 0.279	0.426	0.107	0.961	0.200	0.944	0.196
1.60 : 162.839	0.967 ± 0.146	0.949 ± 0.196	1.027 ± 0.150	1.031 ± 0.206	0.968 ± 0.130	0.983 ± 0.128	0.416	0.103	0.391	0.112	0.934	0.194
1.60 : 164.836	1.003 ± 0.328	0.941 ± 0.157	0.949 ± 0.161	0.922 ± 0.152	1.055 ± 0.124	0.992 ± 0.270	0.399	0.101	0.896	0.193	0.473	0.131
1.61 : 160.842	0.925 ± 0.181	0.874 ± 0.192	0.963 ± 0.166	0.975 ± 0.311	0.972 ± 0.103	1.026 ± 0.190	0.110	0.035	0.236	0.077	0.841	0.181
1.71 : 201.038	0.981 ± 0.073	1.024 ± 0.078	0.979 ± 0.132	1.007 ± 0.188	0.919 ± 0.128	0.927 ± 0.098	0.209	0.052	0.705	0.166	0.700	0.164
1.71 : 89.024	0.998 ± 0.213	1.009 ± 0.331	0.979 ± 0.189	1.036 ± 0.329	0.888 ± 0.229	0.867 ± 0.208	0.549	0.127	0.674	0.151	0.160	0.074
1.77 : 335.050	1.030 ± 0.205	0.873 ± 0.380	1.016 ± 0.300	1.125 ± 0.357	0.992 ± 0.210	0.972 ± 0.152	0.631	0.142	0.946	0.196	0.267	0.096
1.78 : 167.021	1.011 ± 0.116	0.924 ± 0.281	1.004 ± 0.156	1.043 ± 0.236	0.994 ± 0.128	1.006 ± 0.099	0.268	0.070	0.179	0.060	0.083	0.050
1.86 : 191.020	0.774 ± 0.180	0.880 ± 0.364	1.065 ± 0.200	0.904 ± 0.286	0.958 ± 0.382	0.840 ± 0.270	0.057	0.022	0.879	0.187	**0.331**	0.109
1.87 : 128.035	0.689 ± 0.099	0.714 ± 0.211	0.729 ± 0.342	0.796 ± 0.349	0.580 ± 0.114	0.556 ± 0.166	0.869	0.185	**0.331**	0.095	0.364	0.113
2.26 : 151.026	0.827 ± 0.318	1.013 ± 0.426	0.957 ± 0.239	1.000 ± 0.285	1.012 ± 0.343	0.968 ± 0.346	0.869	0.187	0.331	0.097	0.364	0.115
2.49 : 117.019	0.821 ± 0.158	0.798 ± 0.204	0.880 ± 0.226	1.073 ± 0.498	0.779 ± 0.121	0.751 ± 0.084	0.075	0.028	**0.996**	0.202	0.145	0.065
2.55 : 281.036	0.979 ± 0.133	1.019 ± 0.207	1.075 ± 0.255	1.147 ± 0.281	0.952 ± 0.344	0.994 ± 0.173	0.184	0.046	0.161	0.052	0.932	0.193

International Journal of
Molecular Sciences

MDPI

2.66 : 180.067	0.934 ± 0.228	0.869 ± 0.205	0.911 ± 0.199	1.092 ± 0.246	0.925 ± 0.204	1.056 ± 0.160	0.351	0.090	0.016	0.031	0.013	0.017
2.70 : 130.087	0.993 ± 0.309	0.968 ± 0.294	0.936 ± 0.235	1.062 ± 0.202	0.895 ± 0.244	0.926 ± 0.170	0.458	0.112	**0.252**	0.080	0.258	0.094
2.95 : 103.040	0.779 ± 0.648	0.603 ± 0.318	1.391 ± 0.740	0.840 ± 0.356	0.910 ± 0.337	0.711 ± 0.280	0.012	0.006	0.000	0.007	0.281	0.102
4.28 : 115.040	0.899 ± 0.146	0.846 ± 0.232	1.018 ± 0.326	1.070 ± 0.292	0.927 ± 0.183	0.934 ± 0.183	0.210	0.054	0.960	0.198	0.570	0.146
5.70 : 147.045	0.918 ± 0.214	0.887 ± 0.281	0.960 ± 0.219	1.076 ± 0.218	0.908 ± 0.133	0.923 ± 0.112	0.155	0.043	**0.292**	0.088	0.147	0.070
5.84 : 164.072	0.905 ± 0.147	0.822 ± 0.288	0.916 ± 0.248	1.030 ± 0.237	0.897 ± 0.156	0.878 ± 0.100	0.388	0.097	0.919	0.194	0.116	0.059
6.25 : 117.056	0.688 ± 0.195	0.600 ± 0.291	1.219 ± 0.934	1.282 ± 1.264	0.738 ± 0.273	0.742 ± 0.217	0.051	0.020	0.263	0.086	0.464	0.130
6.34 : 188.986	1.383 ± 1.031	0.878 ± 0.551	0.816 ± 0.485	0.947 ± 0.679	0.730 ± 0.355	0.671 ± 0.339	0.441	0.110	0.253	0.082	0.211	0.083
7.27 : 172.991	0.949 ± 0.991	1.091 ± 0.801	1.189 ± 1.302	0.965 ± 0.680	0.844 ± 0.351	0.869 ± 0.401	0.949	0.198	**0.482**	0.125	0.622	0.156
7.61 : 203.083	1.026 ± 0.209	0.887 ± 0.304	0.953 ± 0.172	1.072 ± 0.222	0.965 ± 0.153	0.944 ± 0.141	0.625	0.140	**0.676**	0.153	0.007	0.009
8.47 : 291.030	1.278 ± 0.137	1.049 ± 0.328	1.192 ± 0.261	1.195 ± 0.308	1.265 ± 0.239	1.068 ± 0.360	0.935	0.194	0.008	0.022	0.086	0.052
8.48 : 212.002	0.926 ± 0.399	0.974 ± 0.539	1.173 ± 0.471	1.152 ± 0.585	0.922 ± 0.700	0.835 ± 0.462	0.316	0.080	0.893	0.191	0.969	0.202
8.50 : 254.928	1.077 ± 0.309	0.958 ± 0.308	1.140 ± 0.321	1.078 ± 0.257	0.945 ± 0.284	1.011 ± 0.212	0.272	0.072	0.218	0.073	0.233	0.089
8.66 : 129.056	0.957 ± 0.139	0.838 ± 0.220	1.059 ± 0.169	1.110 ± 0.417	0.878 ± 0.210	0.883 ± 0.241	0.032	0.011	0.200	0.064	0.221	0.085
8.94 : 178.051	0.695 ± 0.269	0.553 ± 0.328	1.133 ± 0.753	1.008 ± 0.773	0.680 ± 0.619	0.742 ± 0.638	0.231	0.059	0.402	0.118	0.274	0.098
8.98 : 263.103	0.747 ± 0.409	0.854 ± 0.850	1.349 ± 0.675	0.950 ± 0.440	0.925 ± 1.001	0.684 ± 0.329	0.074	0.026	0.225	0.075	0.403	0.120
9.57 : 187.008	1.060 ± 1.086	0.901 ± 1.352	1.019 ± 0.604	0.919 ± 0.562	0.797 ± 0.896	0.667 ± 0.529	0.420	0.105	**0.245**	0.079	0.606	0.154
11.19 : 171.066	1.037 ± 0.416	1.010 ± 0.402	0.975 ± 0.335	1.001 ± 0.232	1.086 ± 0.221	1.214 ± 0.370	0.272	0.074	0.422	0.121	0.481	0.133
11.73 : 617.738	0.877 ± 0.448	0.932 ± 0.359	0.980 ± 0.578	1.063 ± 0.652	0.760 ± 0.245	0.909 ± 0.497	0.719	0.151	0.376	0.107	0.928	0.191
11.97 : 187.097	0.863 ± 0.173	0.966 ± 0.365	0.780 ± 0.192	0.964 ± 0.185	0.818 ± 0.144	0.849 ± 0.241	0.675	0.150	0.009	0.024	0.116	0.061
12.16 : 383.153	1.153 ± 0.700	1.248 ± 0.886	0.866 ± 0.426	0.875 ± 0.354	0.848 ± 0.320	0.897 ± 0.394	0.473	0.116	0.555	0.138	0.959	0.200
12.89 : 159.103	0.775 ± 0.237	0.971 ± 0.263	0.884 ± 0.430	1.006 ± 0.379	0.895 ± 0.278	0.805 ± 0.252	0.734	0.159	0.113	0.049	0.053	0.041
13.08 : 413.200	0.905 ± 0.424	0.959 ± 0.223	1.036 ± 0.337	1.104 ± 0.470	1.047 ± 0.539	0.976 ± 0.441	0.794	0.170	0.578	0.142	0.562	0.144
14.72 : 369.174	0.767 ± 0.665	0.776 ± 0.449	1.088 ± 0.655	1.097 ± 0.748	0.823 ± 0.483	0.884 ± 0.318	0.567	0.129	0.671	0.150	0.693	0.163
14.79 : 367.158	1.042 ± 0.435	1.155 ± 0.488	1.055 ± 0.534	1.006 ± 0.471	0.986 ± 0.421	0.982 ± 0.380	0.889	0.191	0.532	0.136	0.276	0.100
14.82 : 510.253	0.972 ± 0.144	0.945 ± 0.187	0.887 ± 0.279	0.927 ± 0.566	1.111 ± 0.472	1.154 ± 0.601	0.217	0.056	0.869	0.185	0.914	0.189
14.88 : 397.205	0.912 ± 0.308	0.894 ± 0.245	0.871 ± 0.399	0.952 ± 0.303	1.068 ± 0.599	0.929 ± 0.389	0.835	0.176	0.600	0.144	0.145	0.067
15.84 : 512.268	1.253 ± 0.507	0.816 ± 0.751	0.874 ± 0.499	1.165 ± 0.821	0.849 ± 0.464	0.726 ± 0.502	0.598	0.131	0.195	0.062	0.051	0.033
16.39 : 185.118	1.039 ± 0.469	1.137 ± 0.874	0.798 ± 0.473	0.919 ± 0.425	0.845 ± 0.332	0.719 ± 0.209	0.295	0.079	0.791	0.170	0.226	0.087
16.64 : 221.118	0.964 ± 0.045	1.002 ± 0.084	0.956 ± 0.039	0.962 ± 0.110	0.990 ± 0.102	0.971 ± 0.066	0.606	0.135	0.699	0.163	0.600	0.152
17.09 : 187.134	0.771 ± 0.408	0.746 ± 0.230	1.162 ± 0.726	0.942 ± 0.378	0.909 ± 0.411	0.732 ± 0.271	0.247	0.063	0.437	0.123	0.586	0.148
17.16 : 376.225	0.871 ± 0.234	0.888 ± 0.229	1.018 ± 0.218	1.081 ± 0.246	1.007 ± 0.311	0.848 ± 0.163	0.075	0.031	0.563	0.140	0.052	0.037
18.19 : 512.299	0.712 ± 0.324	0.896 ± 0.254	0.866 ± 0.513	1.047 ± 0.545	0.771 ± 0.271	0.930 ± 0.499	0.829	0.174	0.013	0.030	0.748	0.170

18.28 : 378.241	0.825 ± 0.266	0.957 ± 0.144	1.069 ± 0.220	0.969 ± 0.150	0.995 ± 0.201	0.875 ± 0.145	0.118	0.039	0.524	0.135	**0.001**	0.007
18.37 : 586.315	0.433 ± 0.168	0.596 ± 0.272	0.655 ± 0.270	0.826 ± 0.618	0.609 ± 0.537	0.478 ± 0.260	0.193	0.048	**0.403**	0.120	0.208	0.081
18.63 : 538.315	0.682 ± 0.201	0.773 ± 0.164	1.020 ± 0.697	0.909 ± 0.485	0.761 ± 0.290	0.922 ± 0.293	0.476	0.118	0.079	0.044	0.060	0.044
18.87 : 194.082	0.998 ± 0.114	0.906 ± 0.151	0.948 ± 0.174	0.970 ± 0.155	1.051 ± 0.201	0.994 ± 0.128	0.343	0.088	**0.335**	0.099	0.545	0.139
18.97 : 500.278	1.027 ± 0.280	0.936 ± 0.203	1.192 ± 0.444	1.104 ± 0.387	1.315 ± 0.302	1.138 ± 0.381	0.285	0.076	0.090	0.046	0.653	0.159
18.98 : 476.278	1.211 ± 0.669	1.065 ± 0.334	0.997 ± 0.486	1.083 ± 0.461	1.302 ± 0.475	1.199 ± 0.522	0.399	0.099	0.849	0.183	0.441	0.122
19.00 : 612.331	0.508 ± 0.254	0.686 ± 0.339	0.815 ± 0.480	0.736 ± 0.370	0.543 ± 0.258	0.456 ± 0.173	0.075	0.030	**0.800**	0.172	0.078	0.048
19.06 : 588.331	0.649 ± 0.244	0.648 ± 0.289	0.796 ± 0.198	0.760 ± 0.249	0.699 ± 0.211	0.604 ± 0.187	0.134	0.041	**0.173**	0.058	0.645	0.157
19.17 : 564.331	0.802 ± 0.177	0.852 ± 0.188	0.842 ± 0.187	0.869 ± 0.240	0.809 ± 0.167	0.723 ± 0.170	0.440	0.108	0.815	0.176	0.331	0.107
19.50 : 452.278	0.936 ± 0.173	0.980 ± 0.221	1.072 ± 0.293	1.044 ± 0.348	1.039 ± 0.355	1.005 ± 0.371	0.768	0.163	0.699	0.161	0.701	0.166
19.61 : 590.346	0.470 ± 0.128	0.597 ± 0.279	0.750 ± 0.350	0.719 ± 0.413	0.532 ± 0.141	0.509 ± 0.269	0.092	0.033	0.815	0.174	0.283	0.104
19.71 : 311.222	1.008 ± 0.585	1.164 ± 0.514	1.224 ± 0.605	1.154 ± 0.850	1.131 ± 0.530	1.168 ± 0.616	0.965	0.202	0.783	0.168	0.552	0.141
19.76 : 540.331	0.926 ± 0.108	0.932 ± 0.117	0.970 ± 0.102	0.980 ± 0.151	0.963 ± 0.064	0.887 ± 0.059	0.373	0.093	0.123	0.050	0.059	0.043
19.93 : 478.294	1.087 ± 0.449	1.312 ± 0.487	1.037 ± 0.519	1.054 ± 0.472	1.106 ± 0.417	1.168 ± 0.413	0.487	0.121	0.161	0.054	0.460	0.128
20.04 : 566.347	0.756 ± 0.136	0.855 ± 0.229	0.925 ± 0.192	0.897 ± 0.231	0.784 ± 0.163	0.718 ± 0.194	0.043	0.017	**0.883**	0.189	0.108	0.057
20.10 : 436.283	0.701 ± 0.207	0.729 ± 0.271	0.763 ± 0.262	0.775 ± 0.350	0.737 ± 0.347	0.493 ± 0.203	0.117	0.037	0.049	0.041	0.015	0.019
20.28 : 524.335	0.661 ± 0.122	0.708 ± 0.190	0.869 ± 0.230	0.951 ± 0.553	0.634 ± 0.201	0.522 ± 0.184	0.001	0.002	0.338	0.101	0.095	0.056
20.33 : 526.350	0.642 ± 0.116	0.708 ± 0.209	0.803 ± 0.246	0.957 ± 0.669	0.662 ± 0.131	0.581 ± 0.143	0.046	0.019	0.396	0.114	0.147	0.069
20.37 : 305.187	0.996 ± 0.182	0.986 ± 0.166	0.962 ± 0.211	0.917 ± 0.137	1.016 ± 0.158	0.966 ± 0.163	0.672	0.148	0.206	0.067	0.840	0.179
20.43 : 233.154	0.899 ± 0.120	0.957 ± 0.169	0.950 ± 0.180	0.930 ± 0.189	0.803 ± 0.169	0.975 ± 0.115	0.646	0.144	0.010	0.028	0.008	0.011
20.60 : 554.346	0.771 ± 0.309	0.751 ± 0.244	1.141 ± 0.377	1.028 ± 0.359	0.910 ± 0.147	0.874 ± 0.315	0.009	0.004	0.202	0.065	0.795	0.176
20.61 : 592.361	0.595 ± 0.122	0.762 ± 0.331	0.770 ± 0.308	0.808 ± 0.389	0.641 ± 0.192	0.558 ± 0.217	0.173	0.044	0.817	0.178	0.078	0.046
20.80 : 552.366	0.626 ± 0.162	0.727 ± 0.272	0.754 ± 0.214	0.838 ± 0.464	0.577 ± 0.164	0.533 ± 0.170	0.019	0.007	0.687	0.157	0.333	0.111
20.94 : 235.170	1.008 ± 0.171	1.089 ± 0.170	1.023 ± 0.186	1.031 ± 0.169	1.028 ± 0.159	1.041 ± 0.149	0.930	0.193	0.210	0.071	0.455	0.126
21.15 : 480.309	1.303 ± 0.409	0.853 ± 0.429	1.267 ± 0.506	1.141 ± 0.222	1.343 ± 0.399	1.135 ± 0.382	0.328	0.084	0.000	0.004	0.033	0.026
21.83 : 483.368	1.309 ± 0.385	1.211 ± 0.436	1.181 ± 0.532	1.117 ± 0.482	1.261 ± 0.448	1.184 ± 0.438	0.610	0.136	0.258	0.084	0.950	0.198
21.85 : 568.362	0.883 ± 0.169	0.904 ± 0.121	1.001 ± 0.208	1.026 ± 0.256	0.987 ± 0.163	0.867 ± 0.188	0.228	0.057	0.309	0.092	0.040	0.031
21.98 : 612.387	1.232 ± 0.607	1.017 ± 0.137	1.041 ± 0.159	1.050 ± 0.146	1.244 ± 0.504	0.941 ± 0.118	0.961	0.200	0.021	0.033	0.012	0.015
21.99 : 585.352	1.270 ± 0.404	1.018 ± 0.174	1.034 ± 0.219	1.024 ± 0.123	1.233 ± 0.356	0.947 ± 0.107	0.791	0.168	0.032	0.037	0.169	0.076
22.00 : 652.323	1.073 ± 0.269	0.941 ± 0.401	1.070 ± 0.084	1.009 ± 0.155	1.312 ± 0.358	0.900 ± 0.116	0.465	0.114	0.000	0.002	0.001	0.006
22.01 : 560.331	1.216 ± 0.375	0.997 ± 0.239	1.080 ± 0.214	1.040 ± 0.137	1.164 ± 0.212	0.944 ± 0.185	0.938	0.196	0.000	0.006	0.034	0.028
22.18 : 464.314	0.709 ± 0.154	0.691 ± 0.187	0.820 ± 0.163	0.839 ± 0.392	0.722 ± 0.380	0.551 ± 0.173	0.039	0.015	0.060	0.043	**0.245**	0.093
22.67 : 271.228	0.837 ± 0.130	0.799 ± 0.208	1.033 ± 0.241	0.909 ± 0.290	0.930 ± 0.255	0.755 ± 0.167	0.247	0.065	**0.004**	0.015	0.558	0.143

International Journal of Molecular Sciences

MDPI

22.67 : 277.217	0.798 ± 0.380	0.740 ± 0.677	0.955 ± 0.707	0.700 ± 0.311	1.445 ± 1.565	1.003 ± 0.998	0.382	0.095	0.022	0.035	0.848	0.183
23.17 : 227.201	0.806 ± 0.331	0.988 ± 0.571	1.224 ± 0.665	0.678 ± 0.193	1.155 ± 0.684	0.885 ± 0.392	0.863	0.179	**0.042**	0.039	0.038	0.030
23.44 : 303.233	1.006 ± 0.371	0.884 ± 0.349	1.092 ± 0.371	0.852 ± 0.353	1.038 ± 0.403	0.797 ± 0.366	0.844	0.178	0.001	0.011	0.596	0.150
23.68 : 485.282	1.044 ± 0.378	1.039 ± 0.393	1.020 ± 0.373	0.886 ± 0.231	1.092 ± 0.301	1.087 ± 0.527	0.623	0.138	0.366	0.105	0.757	0.172
23.80 : 421.332	1.254 ± 0.718	1.053 ± 0.471	1.039 ± 0.343	0.988 ± 0.411	1.097 ± 0.381	1.130 ± 0.282	0.724	0.155	**0.348**	0.103	0.399	0.119
23.84 : 559.473	0.824 ± 0.483	0.842 ± 0.676	1.211 ± 0.801	0.708 ± 0.251	1.342 ± 1.102	0.952 ± 0.892	0.788	0.166	0.008	0.020	0.446	0.124
23.88 : 279.233	0.873 ± 0.310	0.764 ± 0.461	1.124 ± 0.531	0.784 ± 0.222	1.161 ± 0.645	0.904 ± 0.582	0.604	0.133	0.001	0.013	0.896	0.187
23.91 : 375.275	1.578 ± 1.207	1.253 ± 0.763	1.219 ± 1.113	1.118 ± 0.484	1.295 ± 0.870	1.483 ± 1.139	0.788	0.164	**0.846**	0.181	0.725	0.168
24.23 : 445.332	1.215 ± 0.609	1.267 ± 0.544	0.923 ± 0.470	0.890 ± 0.429	0.992 ± 0.310	0.895 ± 0.255	0.263	0.069	0.647	0.146	0.299	0.106
24.58 : 473.282	1.061 ± 0.314	0.989 ± 0.106	0.984 ± 0.225	1.111 ± 0.221	1.020 ± 0.140	0.995 ± 0.257	0.867	0.183	0.703	0.164	0.052	0.039
24.71 : 537.416	1.289 ± 0.617	1.113 ± 0.305	0.973 ± 0.390	1.008 ± 0.438	1.138 ± 0.424	0.965 ± 0.262	0.476	0.120	0.208	0.069	0.391	0.117
24.80 : 299.259	0.917 ± 0.289	0.903 ± 0.239	1.314 ± 0.423	1.233 ± 0.547	1.009 ± 0.540	0.917 ± 0.198	0.022	0.009	**0.497**	0.127	0.886	0.185
25.31 : 255.233	0.723 ± 0.329	0.783 ± 0.425	1.208 ± 0.691	0.727 ± 0.127	1.034 ± 0.418	0.845 ± 0.379	0.251	0.067	**0.006**	0.019	0.051	0.035
25.64 : 281.249	0.846 ± 0.384	0.757 ± 0.417	1.212 ± 0.513	0.784 ± 0.166	1.122 ± 0.517	0.841 ± 0.397	0.321	0.082	0.000	0.009	0.234	0.091

MI: 60% maximal inspiratory pressure (MIP); HI: ~90% MIP; LMM: Linear Mixed Model; p: p-values for the LMM; q: Adjusted p-values for the false discovery rate (FDR); G: Group main effect; T: Time main effect; *G*T*: *Group*Time* interaction. Bold values indicate significant main effects adjusted for the FDR of 0.2 at the significance level of 5%. Data were transformed by Box and Cox procedure before analysis and presented in their original scale. Analyzes were conducted in the SPSS software, version 25.0.

International Journal of *Molecular Sciences*

MDPI

Table S4. Annotation parameters of the compound significantly different between or within-groups obtained by UHPLC-HRMS-based metabolomics.

Compound	LMM effects	HMDB ID	Chemical Formula	Monoisotopic Mass	Ion	*m/z*	Matched fragments (*m/z*) and mass error (ppm)
3-Methylsuberic acid	Time	HMDB0059783	C9H16O4	188.1049	[M-H]-	187.0973	97.0660 (1); 125.0975 (2); 187.0986 (5); 123.0825 (8); 169.0886 (9).
Arachidonic acid	Time	HMDB0001043	C20H32O2	304.2402	[M-H]-	303.2331	59.0136 (5); 205.1957 (0); 231.2111 (0); 259.2435 (4); 285.2233 (5); 303.2323 (2).
Valine	Interaction	HMDB0000883 HMDB0250806	C5H11NO2	117.0790	[M-H]-	116.0712	116.0732 (8).
Hydroxyhexadecanoic acid*	Time	HMDB0112184 HMDB0112186 HMDB0112188 HMDB0112189	C16H32O3	272.2351	[M-H]-	271.2274	55.0185 (7); 225.2217 (3); 253.2193 (8); 271.2273 (2).
Linoleic acid	Time	HMDB0000673	C18H32O2	280.2402	[M-H]-	279.2330	279.2325 (1); 280.2359 (2); 281.2375 (1).
Linolenelaidic acid	Time	HMDB0030964	C18H30O2	278.2246	[M-H]-	277.2170	241.1966 (0); 259.2021 (0); 277.2165 (0).
LysoPC(0:0/16:0)	Interaction	HMDB0240262	C24H50NO7P	495.3325	[M+H]+	496.3404	57.0703 (7); 60.0811 (5); 71.0861 (8); 81.0693 (7); 86.0969 (6); 95.0855 (0); 97.1017 (5); 98.9843 (1); 104.1073 (3); 125.1321 (3); 184.074 (4); 258.1098 (1); 313.2727 (3); 478.3293 (0); 496.3403 (1).
LysoPC(14:0/0:0)	Time	HMDB0010379	C22H46NO7P	467.3012	[M+HCOO]-	512.2996	224.0686 (3); 227.2012 (2); 452.2783 (0).
LysoPC(16:0/0:0)	Time	HMDB0010382	C24H50NO7P	495.3325	[2M+H]+	991.6728	184.0736 (1); 496.3407 (1).
LysoPC(18:2/0:0)	Interaction	HMDB0010386	C26H50NO7P	519.3325	[2M+H]+	1039.6728	86.0970 (7); 104.1069 (1); 124.999 (6); 146.9823 (3); 184.0735 (1); 258.1117 (6); 502.3304 (2); 520.3396 (0); 542.3205 (2).
LysoPC(P-16:0/0:0)	Group	HMDB0010407	C24H50NO6P	479.3376	[M+HCOO]-	524.3358	78.9590 (1); 168.043 (1); 224.0694 (0); 239.237 (4); 464.3135 (2).
LysoPE(18:0/0:0)	Interaction	HMDB0011130	C23H48NO7P	481.3168	[M-H]-	480.3094	152.9957 (1); 214.0497 (5); 283.2638 (2); 480.3107 (2).

LysoPE(P-16:0/0:0)	Interaction	HMDB0011152	C21H44NO6P	437.2906	[M-H]-	436.2821	78.9591 (1); 122.0007 (4); 122.9858 (5); 137.0016 (5); 140.012 (1); 152.9957 (1); 196.0365 (8); 375.2289 (5); 436.2824 (2).
Oleic acid	Time	HMDB0000207	C18H34O2	282.2559	[M-H]-	281.2489	281.2485 (0); 282.2520 (0).
o-Tyrosine	Time/Interaction	HMDB0006050	C9H11NO3	181.0739	[M-H]-	180.0667	72.0091 (0); 74.0255 (9); 93.0340 (3); 107.0500 (2); 119.0501 (1); 134.0609 (1); 136.0771 (2); 163.0403 (1); 180.0660 (3).
Palmitic acid	Time	HMDB0000220	C16H32O2	256.2402	[M-H]-	255.2331	237.2239 (10); 255.2328 (1); 256.2354 (1).
Sphingosine 1-phosphate	Interaction	HMDB0000277	C18H38NO5P	379.2488	[M-H]-	378.2411	78.9590 (1); 378.2403 (3).
Tryptophan	Interaction	HMDB0000929 HMDB0013609	C11H12N2O2	204.0899	[M+H]+	205.0971	91.0541 (2); 115.0548 (10); 117.0576 (7); 118.0654 (3); 130.0652 (5); 132.0813 (1); 140.0488 (3); 142.0653 (2); 143.0725 (7); 144.081 (3); 146.0605 (5); 147.0637 (5); 159.0917 (2); 188.0713 (1).
Tryptophan	Interaction	HMDB0013609 HMDB0000929	C11H12N2O2	204.0899	[M-H]-	203.0828	116.0505 (1); 117.0536 (5); 142.0661 (6); 159.0917 (8); 186.0567 (2); 204.0859 (0).
Unknown	Interação	-	-	-	Positive	Unfragmented	-
Unknown	Interação	-	-	-	Positive	Unfragmented	-
Unknown	Interação	-	-	-	Positive	Unfragmented	-
Unknown	Interação	-	-	-	Positive	550.3883	-
Unknown	Interação	-	-	-	Positive	Unfragmented	-
Unknown	Interaction	-	-	-	Negative	Unfragmented	-
Unknown	Interaction	-	-	-	Negative	152.8837	-
Unknown	Interaction	-	-	-	Negative	112.9856	-
Unknown	Interaction	-	-	-	Negative	Unfragmented	-
Unknown	Interaction	-	-	-	Positive	Unfragmented	-
Unknown	Interaction	-	-	-	Positive	Unfragmented	-

Unknown	Interaction	-	-	-	Positive	Unfragmented	-
Unknown	Interaction	-	-	-	Positive	Unfragmented	-
Unknown	Interaction	-	-	-	Positive	Unfragmented	-
Unknown	Interaction	-	-	-	Positive	Unfragmented	-
Unknown	Interaction	-	-	-	Positive	Unfragmented	-
Unknown	Time	-	-	-	Negative	96.9600	-
Unknown	Time	-	-	-	Negative	103.0398	-
Unknown	Time	-	-	-	Negative	585.3508	-
Unknown	Time	-	-	-	Negative	Unfragmented	-
Unknown	Time	-	-	-	Negative	559.4726	-
Unknown	Time	-	-	-	Negative	291.0310	-
Unknown	Time	-	-	-	Positive	Unfragmented	-
Unknown	Time	-	-	-	Positive	274.2742	-
Unknown	Time	-	-	-	Positive	209.1022	-
Unknown	Time	-	-	-	Positive	Unfragmented	-
Unknown	Time/Interaction	-	-	-	Positive	Unfragmented	-
Unknown	Time/Interaction	-	-	-	Negative	154.9180	-
Unknown	Time/Interaction	-	-	-	Negative	233.1538	-
Unknown	Time/Interaction	-	-	-	Negative	612.3843	-
Unknown	Time/Interaction	-	-	-	Negative	652.3241	-

LMM: Linear mixed model effects; *Hydroxylated isomers.

International Journal of *Molecular Sciences*

MDPI

Table S5. Summary of the Metabolite Set Enrichment Analysis and altered pathways Pre to Post-inspiratory muscle training.

Pathway	Library	Total	Expected	Hits	P-value	FDR	Decision	Enrichment ratio*
Biosynthesis of unsaturated fatty acids	KEGG	36	0.328	4	0.0002	0.0063	SIG	12.20
Aminoacyl-tRNA biosynthesis	KEGG	48	0.438	4	0.0007	0.0125	SIG	9.13
Ketone body metabolism	SMPDB	13	0.241	3	0.0014	0.0188	SIG	12.45
Butanoate metabolism	KEGG	15	0.137	2	0.0076	0.0250	SIG	14.60
Citrate cycle	KEGG	20	0.182	2	0.0134	0.0313	SIG	10.99
Propanoate metabolism	KEGG	23	0.21	2	0.0175	0.0375	SIG	9.52
Fatty acid biosynthesis	SMPDB	35	0.649	3	0.0244	0.0438	SIG	4.62
Alanine, aspartate and glutamate metabolism	KEGG	28	0.255	2	0.0255	0.0500	SIG	7.84
Alpha linolenic acid and linoleic acid metabolism	SMPDB	19	0.353	2	0.0462	0.0563	SIG	5.67
Butyrate metabolism	SMPDB	19	0.353	2	0.0462	0.0625	SIG	5.67
Valine, Leucine and Isoleucine Degradation	SMPDB	60	1.11	3	0.0949	0.0688	NS	2.70
Phospholipid biosynthesis	SMPDB	29	0.538	2	0.0984	0.0750	NS	3.72
Arginine and Proline Metabolism	SMPDB	53	0.983	2	0.2580	0.0813	NS	2.03
Warburg effect	SMPDB	58	1.08	2	0.2930	0.0875	NS	1.85
Arachidonic acid metabolism	SMPDB	69	1.28	2	0.3700	0.0938	NS	1.56
Tyrosine metabolism	SMPDB	72	1.34	2	0.3910	0.1000	NS	1.49

*Pathway enrichment ratio is computed by Hits/Expected hits; FDR: False discovery rate of 0.1.

International Journal of *Molecular Sciences*

MDPI

Table S6 - Metabolite-metabolite networks sub-information table.

KEGG ID	Label	Degree of centrality	Betweenness	Technique
C00002	Adenosine triphosphate	12	32.73	Not annotated
C00007	Oxygen	10	25.99	Not annotated
C00158	Citrate	10	23.83	^{1}H NMR
C00073	Methionine	10	15.83	^{1}H NMR
C00004	NADH	9	19.60	Not annotated
C00219	Arachidonic acid	9	16.09	UHPLC-HRMS/MS
C03878	Beta-N-Acetylglucosamine	9	13.73	Not annotated
C00042	Succinate	8	21.58	^{1}H NMR
C00078	Tryptophan	8	14.15	UHPLC-HRMS/MS
C00249	Palmitic acid	8	9.14	UHPLC-HRMS/MS
C00183	Valine	8	2.31	UHPLC-HRMS/MS
C00712	Oleic acid	7	3.30	UHPLC-HRMS/MS
C00148	Proline	7	1.15	^{1}H NMR
C01595	Linoleic acid	6	1.20	UHPLC-HRMS/MS
C00218	Methylamine	5	10.19	^{1}H NMR
C00027	Hydrogen peroxide	5	4.96	Not annotated
C01996	O-acetylcholine	5	0.48	^{1}H NMR
C00166	Phenylpyruvic acid	4	21.14	Not annotated
C00207	Acetone	3	4.48	^{1}H NMR
C00356	3-Hydroxy-3-methylglutaryl-CoA	3	1.47	Not annotated
C01089	3-Hydroxybutyrate	3	1.40	^{1}H NMR
C08262	Isovaleric acid	2	0.28	Not annotated
C05984	2-Hydroxybutyrate	1	0.00	^{1}H NMR

KEGG: Kyoto Encyclopedia of Genes and Genomes.

International Journal of *Molecular Sciences*

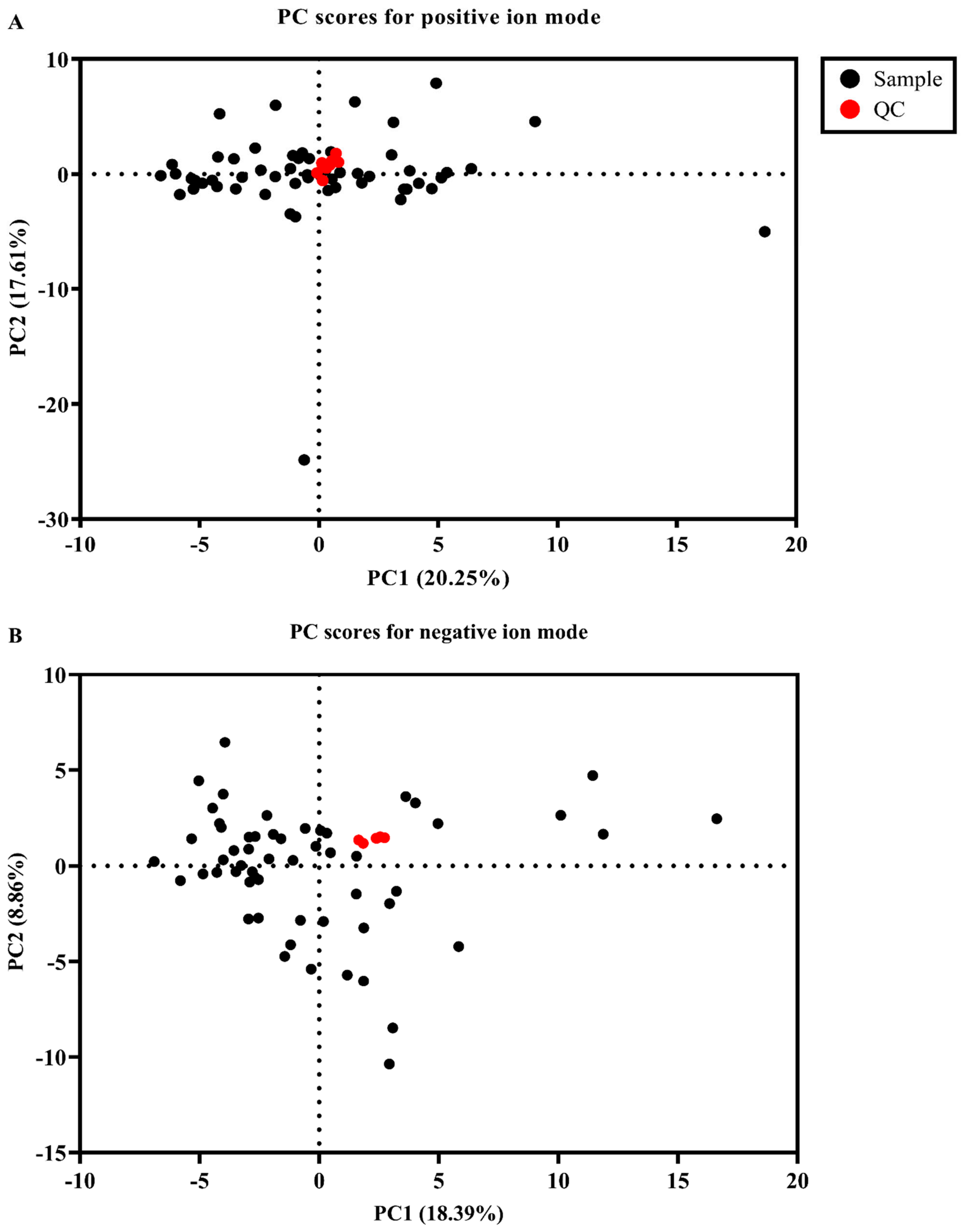

Figure S1. Instrumental stability assessed through quality control samples for the data obtained by UHPLC-HRMS/MS in positive (**A**) and negative (**B**) ionization mode.
